# Chest CT-derived body composition parameters for outcome prediction in sepsis patients with pneumonia

**DOI:** 10.1080/07853890.2025.2584421

**Published:** 2025-11-10

**Authors:** Xin Qiao, Xinyu Li, Qiuyue Wang, Rui Zheng

**Affiliations:** ^a^ Department of Pulmonary and Critical Care Medicine, Shengjing Hospital of China Medical University, Shenyang, China; ^b^ Department of Pulmonary and Critical Care Medicine, The First Hospital of China Medical University, Shenyang, China

**Keywords:** Body composition, chest CT, mortality, MICU admission, sepsis

## Abstract

**Objective:**

We aim to evaluate the potential contribution of chest computed tomography (CT)-derived body composition parameters in predicting adverse events in sepsis patients with pneumonia.

**Methods:**

A retrospective study was conducted on sepsis with pneumonia cases who visited Shengjing Hospital of China Medical University from January 2023 to September 2024. We used chest CT scans to quantify skeletal muscle area (SMA) at the fourth thoracic vertebra (T4) and the first lumbar vertebra (L1) levels, as well as abdominal circumference (AC), subcutaneous adipose tissue (SAT), and intramuscular adipose tissue (IMAT) at the L1 level.

**Results:**

A total of 303 patients (203 men; median age 70 years, interquartile range 63–79) were included in the study. Fully adjusted models identified low SMA_T4_, low SAT_L1_, and high AC_L1_ as independent risk factors for medical intensive care unit (MICU) admission, with odds ratios (ORs) of 0.795, 0.897, and 2.095, respectively. Low SMA_T4_ (OR 0.880, 95% confidence interval [CI] 0.800–0.967, *p* = 0.008) and high AC_L1_ (OR 1.527, 95% CI 1.122–2.079, *p* = 0.007) were both independently associated with in-hospital mortality. High IMAT_L1_ (*β*: −2.360, *p* = 0.003) was associated with a greater decrease in the PaO_2_/FiO_2_ ratio during hospitalization. Models using chest CT-derived body composition parameters to predict MICU admission, septic shock, and in-hospital mortality in patients with sepsis were as effective as sequential organ failure assessment scores.

**Conclusions:**

The assessment of frailty status and visceral obesity, determined by chest CT measurements of low thoracic muscle mass and elevated AC, is independently correlated with an increased risk of admission to the MICU and mortality among sepsis patients with pneumonia. This underscores the significance of CT-derived body composition as a critical imaging biomarker that reflects the physiological reserve of sepsis patients and their associated risk of adverse events.

## Introduction

Sepsis is a serious condition marked by organ dysfunction due to an abnormal response to infection, requiring urgent treatment due to its rapid progression [1]. Within the spectrum of community-acquired sepsis, pneumonia represents a leading infectious burden (accounting for approximately 40%), varying from mild respiratory symptoms to severe disease states requiring hospitalization, admission to the intensive care unit (ICU), mechanical ventilation, and potentially culminating in mortality [2,3]. The early identification of risk factors for adverse outcomes in these patients is crucial for both patient risk stratification and the development of potential treatment strategies. Previous research indicates that elderly patients and individuals with underlying comorbidities, including cardiovascular disease, chronic kidney disease (CKD), chronic obstructive pulmonary disease (COPD), and diabetes, are at an elevated risk of experiencing severe complications [4–7]. Recently, two novel prognostic body composition parameters, sarcopenia (low skeletal muscle mass) and visceral obesity, have been identified as independent predictors of adverse outcomes in patients with sepsis [8,9].

Previous studies have shown that skeletal muscle cross-sectional area (CSA) and visceral fat CSA at the third lumbar vertebra (L3) level, as assessed by abdominal computed tomography (CT), are important predictors of poor prognosis in sepsis patients [8–10]. However, in the largest group of patients with sepsis who have pneumonia, chest CT is more commonly used, providing a convenient and accessible method for assessing body composition. Studies have demonstrated that muscle and fat mass measurements at the fourth thoracic vertebra (T4) or the first lumbar vertebra (L1) levels *via* chest CT exhibit a strong correlation with measurements obtained at the L3 level and hold substantial prognostic value for critically ill patients [11–14]. Nevertheless, the majority of existing studies focus on specific muscle groups, such as the pectoralis major and erector spinae, and are mainly conducted in patient who have contracted coronavirus disease 2019 (COVID-19) or have mixed infection sources. The condition of the thoracic muscles, particularly those muscles directly involved in the respiratory process—such as the pectoral muscles, intercostal muscles, latissimus dorsi, and paraspinal muscles—is well-documented to have a direct impact on respiratory function [15]. Consequently, evaluating the overall muscle mass at the T4 level may provide a more valuable prognostic indicator for sepsis patients with pneumonia. Currently, there is a lack of studies to evaluate the predictive value of chest CT-derived body composition parameters at the T4 and L1 levels in this large subgroup of sepsis patients. Moreover, it is essential to determine which of the T4 and L1 muscle CSA serves as a more effective predictor of adverse events in this condition.

At present, clinical decisions in the management of sepsis mainly rely on vital signs, including blood pressure, respiratory rate, and body temperature, alongside routine laboratory analyses such as lactate levels, inflammatory markers, and indicators of organ function [16]. The Sequential Organ Failure Assessment (SOFA) score integrates this information and has been established as an important clinical tool for evaluating organ dysfunction and predicting short-term mortality in sepsis patients [17]. However, these conventional parameters mainly reflect the acute physiological state and organ dysfunction, providing limited information on the patient’s potential prognostic physical reserve status (e.g. muscle mass and fat distribution). A large body of evidence indicates that CT is a suitable tool for identifying the majority of infectious foci in patients with sepsis or septic shock undergoing medical intensive care [18,19]. While CT plays a role in decision-making for sepsis patients, positive CT findings do not predict patient outcomes [18]. This prompts the question of whether the predictive capacity of CT-derived body composition parameters is comparable to that of the SOFA score. More importantly, can they provide additional prognostic information beyond the conventional clinical and laboratory parameters in order to more comprehensively identify high-risk patients and optimize management strategies?

Therefore, our aim was to investigate whether the body composition parameters from chest CT scans hold predictive value for adverse outcomes in sepsis patients with pneumonia.

## Materials and methods

### Study population

A single-center retrospective cohort study design was employed. The cohort study enrolled sepsis patients who were treated at Shengjing Hospital of China Medical University between January 2023 and September 2024. The diagnostic criteria for sepsis adhered to the Third International Consensus Definitions for Sepsis and Septic Shock (Sepsis-3) [1]. The specific assessment process is as follows: Step 1: Initial screening criteria: Patients admitted to the hospital with an inpatient diagnosis of ‘pneumonia’ and meeting any of the following criteria will be selected from the electronic medical record system: (a) A diagnosis of ‘sepsis’ or ‘septic shock’; (b) Sequential Organ Failure Assessment (SOFA) records exist, and the score is ≥ 2 points. tep 2: Retrospective validation of the Sepsis-3 criteria: (1) Infection confirmation: Pneumonia is identified as the source of infection through chest CT at the time of presentation, along with acute respiratory symptoms and microbiological reports (sputum, bronchoalveolar lavage fluid, etc.) during hospitalization; If microbial evidence is unavailable but the CT images are definitive and antibiotic treatment is effective, these patients will still be included in the analysis. (2) SOFA score calculation: Parameters required for the SOFA score are extracted from the first laboratory report upon admission (blood routine, liver and kidney function, blood gas analysis, etc.) and the first vital sign record (mean arterial pressure, respiratory rate, oxygenation index, Glasgow Coma Scale, etc.). An increase of ≥ 2 points in the SOFA score compared to the baseline (without acute disease state) is defined as organ dysfunction.

**Figure 1.**
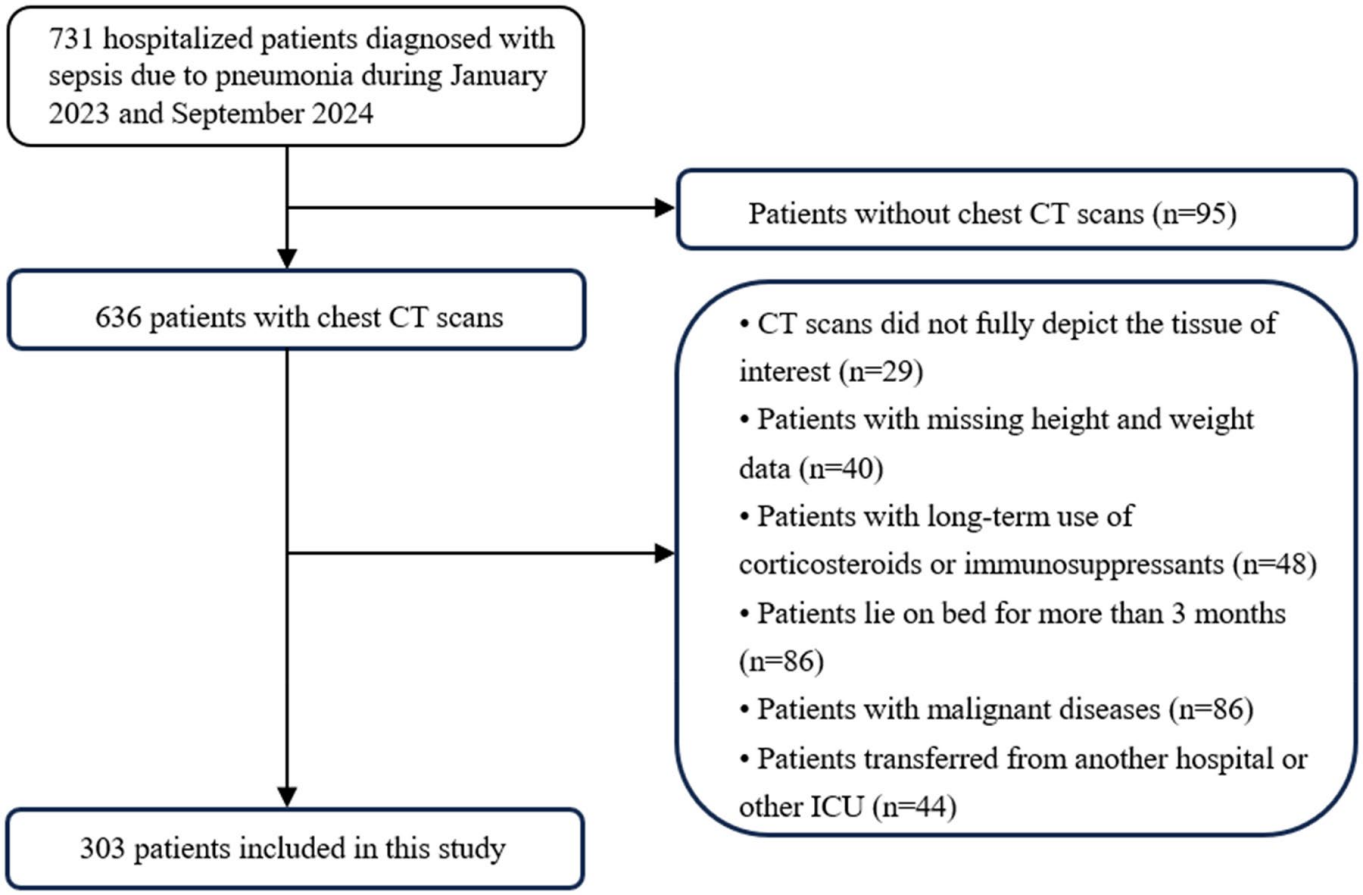
The process of patient selection.

Together, the following criteria are used for patient inclusion: (1) Pneumonia as the source of infection with an increase of ≥2 points in the SOFA score compared to the baseline; (2) a hospitalization duration exceeding 4 days; and (3) performance of a chest CT scan at the time of presentation. The exclusion criteria for patients in this study were defined as follows: (1) absence of CT images in the institutional Picture Archiving and Communication System (PACS) viewer; (2) CT images that were of suboptimal quality, contained artifacts, or failed to adequately represent the tissue of interest; (3) prolonged use of corticosteroids or immunosuppressants, including individuals who have undergone organ transplantation; (4) patients who are long-term bedridden, such as those with cerebrovascular sequelae, spinal cord injury, or myasthenia gravis, resulting in permanent sensory and/or motor deficits; (5) the presence of malignant diseases; and (6) patients transferred from another hospital or ICU. The flowchart for inclusion and exclusion criteria of our cohort is shown in Figure 1. The study has received ethical approval from the Ethics Committee of Shengjing Hospital of China Medical University and the patient has provided written informed consent for participation. Written informed consent was obtained from the individual(s) for the publication of any potentially identifiable images or data included in this article.

### Data collection

Patient-specific data at admission were retrieved from electronic records, including demographics, body mass index (BMI), comorbidities, and laboratory test results. Clinical outcomes, such as medical intensive care unit (MICU) admission, initiation of invasive mechanical ventilation (IMV), occurrence of septic shock, and discharge or mortality, were retrospectively retrieved. The SOFA scores, which range from 0 to 24 points, and the Acute Physiology and Chronic Health Evaluation (APACHE) II scores, which range from 0 to 71 points, were calculated in accordance with methodologies established in a previous study [20].

### CT scan analysis

**Figure 2.**
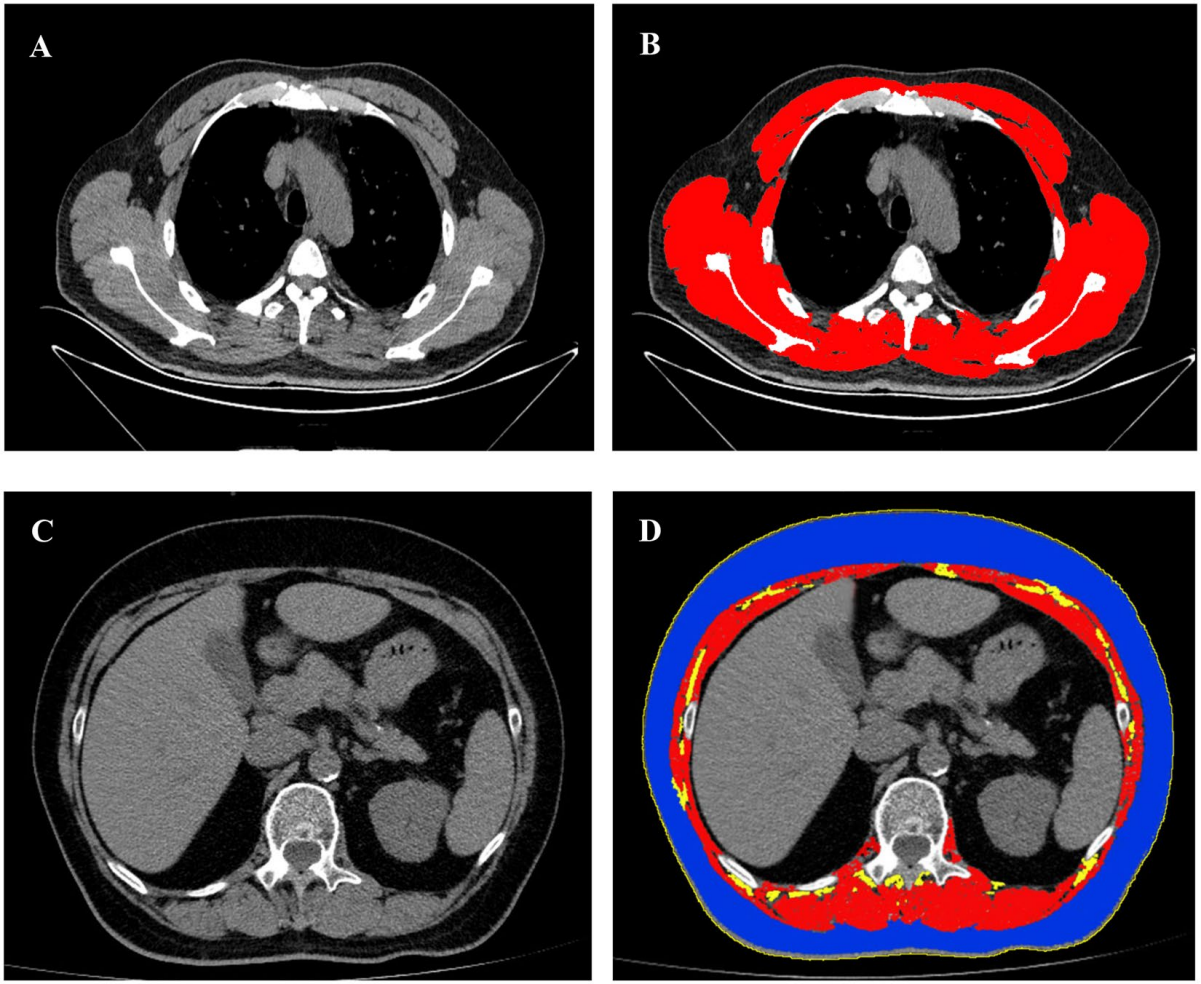
Representative examples of selected CT scan slices at the fourth thoracic vertebra level (A) with demarcated thoracic muscle (B) and at first lumbar vertebra (L1) level (C) with demarcated L1 muscle, subcutaneous adipose tissue, intramuscular adipose tissue, and abdominal circumference (D).

All the included patients underwent 64-slice spiral CT (Toshiba Medical Corporation, Japan) scans at Shengjing Hospital. Scanning conditions included a tube ball voltage of 135 kV, a rotation time of 0.4 s, a window width of 1600, a window position of −600, and a reconstruction layer thickness of 5 mm or less after continuous scanning from the bottom of the lung to the tip of the lung. Under the guidance of two radiologists with 5–10 years of experience in chest imaging, one assessor manually located the T4 and L1 levels using the institutional PACS viewer. As described in previous studies, one trained assessor manually divided adipose tissue and skeletal muscle based on pre-established Hounsfield units (HU) thresholds (skeletal muscle, −29 to 150 HU; subcutaneous adipose tissue [SAT] and intramuscular adipose tissue [IMAT], −190 to −30 HU) [11,21]. Single-slice CT images of each patient were acquired at the T4 and L1 levels for subsequent analysis using ImageJ version 1.8.0 software (NIH, Bethesda, MD). Three trained respiratory physicians calculated skeletal muscle CSA, adipose tissue CSA, and abdominal circumference (AC) by delineating regions of interest with a mouse interface (Figure 2). All evaluators blinded to clinical information and outcomes. The skeletal muscle area at the T4 level (SMA_T4_) encompasses the combined CSA of the pectoralis major, pectoralis minor, intercostals, axillary fossa, arm muscles, latissimus dorsi, teres major, infraspinatus, teres minor, subscapularis, serratus, rhomboids, trapezius, and paraspinal muscles. The skeletal muscle area at the L1 level (SMA_L1_) encompasses the combined CSA of the psoas, erector spinae, quadratus lumborum, transversus abdominis, external and internal oblique, and rectus abdominis muscles. The skeletal muscle index (SMI, cm^2^/m^2^) was calculated as the ratio of muscle area (cm^2^) to height squared (m^2^).

### Statistical analysis

Continuous data are reported as the mean (± standard deviation, SD) or median (interquartile range [IQR]) depending on their statistical distribution, and categorical data are reported as the number of events (percentages). Spearman correlation analysis was used to test the association between SMI_T4_, SMI_L1_, and SMI_L3_. The relationship between potential predictors and the length of hospital stays, as well as the changes in the PaO_2_/FiO_2_ ratio and SOFA scores was assessed using univariate linear regression models. Variables with a *P* value of less than 0.05 were included in the multivariate analysis to assess their relative contributions. The association between potential predictors and the risks of MICU admission, IMV, septic shock, and in-hospital mortality was firstly assessed using univariate binary logistic regression models. Subsequently, we sought to evaluate the outcome discrimination performance of three distinct models, all of which included sex, age, and BMI as covariates, each emphasizing a different set of additional variables. Model 1 incorporated only clinical variables, Model 2 focused solely on body composition, and Model 3 included both clinical variables and body composition. Variable selection for model construction was conducted using multivariable linear regression through backward elimination. Covariate selection for the predictive model should follow the guideline of 15 events per variable (i.e. deaths) [22]. If the number of covariates exceeds this limit, prioritize those with lower *p* values and higher *T* statistics. The covariates satisfying these criteria were subsequently incorporated into a multivariate binary logistic regression analysis, allowing for the calculation of adjusted odds ratios (ORs) and 95% confidence intervals (CIs).

The performance of the obtained models predicting outcomes was assessed using receiver operating characteristic (ROC) curve analysis and area under the curve (AUC). The stability of the predictive model was assessed through five-fold internal cross-validation [23]. The entire research dataset was randomly partitioned into five approximately equal-sized subsets (folds). Subsequently, five sequential rounds of training and validation were performed: during the i-th round (where *i* = 1, 2, 3, 4, 5), data from the remaining four folds (comprising approximately 80% of the samples) were utilized to train the model. The trained model was then employed to predict the risk probability for the data retained in the i-th fold (approximately 20% of the samples, which were designated as the validation set), thereby obtaining the prediction probability for each sample. Utilizing the true outcome status of the validation set samples and the corresponding prediction probabilities, the AUC for each fold was calculated (AUC_fold_i). The arithmetic mean of the five AUC_fold_i values (mean_AUC) was computed to serve as a comprehensive indicator of the model’s performance, reflecting its average discriminative capability across multiple data partitions. After completing all five rounds of cross-validation, the overall AUC based on the combined prediction probabilities from all folds, is calculated. This metric provides the most reliable estimate of the model’s anticipated performance across the entire target population. Comparisons of AUCs were conducted using the DeLong method [24].

Body composition parameters in the cohort were converted into categorical variables based on sex-specific IQRs. Univariate and multivariate binary logistic regressions were used to assess the association of stratified body parameters with adverse outcomes. A two-tailed *P* value below 0.05 indicated statistical significance. All statistical analyses were performed using IBM SPSS Statistics version 19.0 (IBM Corp, Armonk, NY, USA), GraphPad Prism version 8.0 (GraphPad Software, San Diego, CA, USA), and R software version 4.5.1 (R Foundation for Statistical Computing, Vienna, AUS).

## Results

### Cohort description

From the initial cohort of 731 patients recruited for this study, a total of 428 patients were excluded from the analysis and 303 patients were included in the study. The baseline characteristics of participants are presented in Table 1. Among these 303 patients, 203 (67.0%) were men and 100 (33.0%) were women, with an overall median age of 70 years (IQR: 63–79). The median length of hospitalization was 13 days (IQR 10–18), and 182 (60%) patients were admitted to the MICU. The overall in-hospital mortality was 27.4% (83/303 patients). No significant differences in age, sex, or BMI were observed between the MICU group and the non-MICU group. Within the MICU cohort, 80 patients underwent IMV, with a median duration of IMV of 7 days (IQR 3.6–13.5). The MICU group demonstrated significantly elevated levels of white blood cell count (WBC), neutrophil count, D-dimer, and aspartate transaminase (AST). In contrast, they exhibited reduced levels of the PaO_2_/FiO_2_ ratio, albumin (ALB), lymphocyte count, and hemoglobin (Hgb) compared to the non-MICU group (*p* < 0.05).

**Table 1.** General characteristics.

Variables	Total (*N* = 303)	Non-MICU (*N* = 121)	MICU (*N* = 182)	*p* value^a^
**Demographics**				
Age (years)	70 (63–79)	70 (62–79)	70 (64–79)	0.999
Male (*N*, %)	203 (67.0)	83 (68.6)	120 (65.9)	0.361
Height (cm)	170 (160–174)	170 (160–174)	170 (160–175)	0.078
Weight (kg)	66 (58–75)	68 (60–76)	65 (55–75)	0.425
BMI (kg/m^2^)	23.4 (20.8–26.0)	23.9 (21.2–27.1)	23.0 (20.8–25.4)	0.189
**Comorbidities at admission (*N*, %)**				
COPD	27 (8.9)	7 (5.8)	20 (11.0)	0.086
Heart failure	97 (32.0)	22 (18.2)	75 (41.2)	**0.000**
Hypertension	121 (39.9)	47 (38.8)	74 (40.7)	0.423
CKD	86 (28.4)	22 (18.2)	64 (35.2)	**0.001**
Diabetes	91 (30.0)	37 (30.6)	54 (29.7)	0.482
Chronic liver disease	31 (10.2)	4 (3.3)	27 (14.8)	**0.001**
**Laboratory findings**				
WBC (*10^9^/L)	8.5 (6.1–12.5)	7.7 (5.8–10.5)	9.2 (6.8–14.4)	**0.019**
Neutrophil count (*10^9^/L)	7.0 (4.6–10.7)	5.5 (3.9–8.8)	7.6 (5.2–12.9)	
Lymphocyte count (*10^9^/L)	0.8 (0.5–1.3)	1.1 (0.7–1.5)	0.7 (0.4–1.0)	**0.000**
Hgb (g/L)	115.6 ± 24.6	122.5 ± 20.4	110.9 ± 26.0	**0.000**
PLT (*10^9^/L)	185 (135–262)	190 (140–278)	178 (118–254)	0.398
D dimer (ug/mL)	0.71 (0.24–1.86)	0.48 (0.22–1.32)	1.1 (0.4–2.6)	**0.000**
TNI (ug/L)	0.39 (0.01–3.80)	0.03 (0.01–4.5)	0.67 (0.02–3.47)	0.111
ALT (U/L)	25 (16–42)	23 (15–38)	27 (17–45)	0.452
AST (U/L)	26 (18–42)	23 (16–34)	29 (20–47)	**0.006**
GGT (U/L)	41 (22–79)	33 (20–70)	45 (25–81)	0.100
ALB (g/L)	30.8 ± 5.7	32.8 ± 4.9	29.4 ± 5.8	**0.000**
Cr (umol/L)	70 (53–98)	69 (57–87)	72 (51–109)	0.742
CRP (mg/L)	77.4 (23.2–133.8)	60.6 (15.4–114.1)	87.6 (33.8–153.9)	0.067
**Hospitalization data**				
Hospital LOS (days)	13 (10–18)	12 (10–15)	14 (10–19)	**0.005**
MICU LOS (days)	N/A	N/A	12 (8–17)	N/A
IMV (*N*, %)	80 (26.4)	N/A	80 (44.0)	N/A
Length of IMV (days)	N/A	N/A	7.0 (3.6–13.5)	N/A
Septic shock (*N*, %)	78 (25.7)	2 (1.7)	76 (41.8)	**0.000**
Mortality (*N*, %)	83 (27.4)	5 (4.1)	78 (42.9)	**0.000**
APACHE II score	N/A	N/A	13 (10–18)	N/A
SOFA score at admission	2.0 (2.0–4.0)	2 (2–3)	3 (2 –6)	**0.000**
Highest SOFA score	3 (2–9)	2 (2–3)	7 (3–11)	**0.000**
△SOFA score^b^	0 (0–3)	0	1 (0–6)	**0.000**
PaO_2_/FiO_2_ at admission (mmHg)	247.6 (180.0–290.5)	276.2 (236.2–297.0)	215.5 (150.8–277.1)	**0.000**
Lowest PaO_2_/FiO_2_ (mmHg)	184.8 (91.8–276.0)	265.5 (224.0–294.3)	118.2 (65.5–200.9)	**0.000**
△PaO_2_/FiO_2_^c^	0 (−93.1, 0)	0	−49.4 (−122, 0)	0.110
**CT-derived body composition parameters**				
SMA_T4_, cm^2^	144.5 (119.2–173.7)	153.5 (131.6–177.1)	138.2 (111.9–165.9)	**0.009**
SMI_T4_, cm^2^/m^2^	51.1 (43.4–59.7)	54.7 (47.5–62.5)	47.7 (40.9–57.5)	**0.001**
SMA_L1_, cm^2^	92.6 (76.2–113.0)	95.2 (80.8–113.6)	89.0 (70.3–112.9)	0.146
SMI_L1_, cm^2^/m^2^	32.9 (27.3–39.1)	33.6 (28.8–40.3)	32.0 (26.0–38.2)	0.223
SAT_L1_, cm^2^	80.6 (52.5–122.3)	83.2 (54.4–129.4)	78.9 (51.2–116.9)	0.542
AC_L1_, cm	95.4 (88.4–103.3)	94.9 (88.9–101.3)	96.1 (87.4–104.7)	0.373
IMAT_L1_, cm^2^	5.4 (3.2–8.3)	5.1 (3.2–7.7)	5.5 (3.2–8.5)	0.511

Results are presented as median (interquartile range), mean ± standard deviation, or number of events (%). Bold face indicates a *p* value with statistical significance. MICU: medical intensive care unit; cm: centimeter; m: meter; kg: kilogram; BMI: body mass index; COPD: chronic obstructive pulmonary disease; CKD: chronic kidney disease; WBC: white blood cell; Hgb: hemoglobin; PLT: platelet; TNI: troponin I; ALT: alanine aminotransferase; AST: aspartate transaminase; GGT: gamma-glutamyl transferase; ALB: albumin; Cr: creatinine; CRP: C-reactive protein; LOS: length of stay; IMV: invasive mechanical ventilation; APACHE: acute physiology and chronic health evaluation; SOFA: sequential organ failure assessment; PaO_2_: arterial partial pressure of oxygen; FiO_2_: fractional oxygen concentration; CT: computed tomography; SMA_T4_: skeletal muscle area at T4 level; SMI_T4_: skeletal muscle index at T4 level; SMA_L1_: skeletal muscle area at L1 level; SMI_L1_: skeletal muscle index at L1 level; SAT_L1_: subcutaneous adipose tissue at L1 level; AC_L1_: abdominal circumference at L1 level; IMAT_L1_: intramuscular adipose tissue at L1 level.

^a^ *p* values for comparison between MICU versus non-MICU.

^b^ △SOFA score = Highest SOFA score – SOFA score at admission.

^c^ △PaO_2_/FiO_2_ = Lowest PaO_2_/FiO_2_ – PaO_2_/FiO_2_ at admission.

### CT image analyses

As presented in Table 1, patients admitted to the MICU exhibited significantly lower SMA_T4_ (*p* = 0.009) and SMI_T4_ (*p* = 0.001) compared to the non-MICU group. Patients over 80 years old had the lowest median muscle mass, while AC_L1_, IMAT_L1_ and SAT_L1_ were not significantly different from other groups (Supplementary Figure 1A–G). Men had significantly higher muscle mass and AC_L1_ but lower fat mass than women (Supplementary Figure 2A–D). In addition, there was a strong correlation between SMI_T4_ (*r* = 0.656, *p* = 0.000) or SMI_L1_ (*r* = 0.763, *p* = 0.000) and SMI_L3_ (Supplementary Figure 3A–B).

Both SMA_T4_ and SMI_T4_ exhibited a positive correlation with the change of PaO_2_/FiO_2_ ratio, whereas IMAT_L1_ demonstrated a negative correlation (Supplementary Table 1). In a multivariate analysis that adjusted for baseline PaO_2_/FiO_2_ ratio, heart failure, neutrophil count, lymphocyte count, SMA_T4_, and SMI_T4_, IMAT_L1_ remained significantly associated with the change of PaO_2_/FiO_2_ ratio (*β*: −2.360, 95% CI −3.928, −0.791, *p* = 0.003). The univariate linear regression analysis indicated a negative correlation between SMA_T4_ and SMI_T4_ with the change in SOFA score; however, this association lost statistical significance after adjusting for covariates (Supplementary Tables 1 and 2). There was no significant correlation between CT-derived body composition parameters and the length of hospital stay, regardless of whether in univariate or multivariate analysis (Supplementary Tables 1 and 2).

### Risk prediction of adverse events

The unadjusted ORs for each variable concerning admission to the MICU, IMV, septic shock, and mortality, derived from univariate binary logistic regression, are presented in the initial columns of Tables 2 and 3, Supplementary Table 3 and 4, respectively. Details of model building through multivariable linear regression are shown in Supplementary Tables 5–7. In the multivariate logistic regression analysis of Model 2 (which only included body composition parameters based on CT imaging), high AC_L1_ was a significant risk factor for admission to the MICU (OR 2.329, 95% CI 1.618–3.353, *p* < 0.001; Table 2), as well as a significant risk factor for IMV (OR 1.512, 95% CI 1.170–1.954, *p* = 0.002; Supplementary Table 3), septic shock (OR 2.492, 95% CI 1.649–3.767, *p* < 0.001; Supplementary Table 4), and in-hospital mortality (OR 1.501, 95% CI 1.137–1.982, *p* = 0.004; Table 3). At the same time, when evaluating the risk of MICU admission in Model 2, high SMA_T4_ (OR 0.795, 95% CI 0.708–0.893, *p* < 0.001; Table 2) and high SAT_L1_ (OR 0.897, 95% CI 0.829–0.971, *p* = 0.007; Table 2) were identified as independent protective factors.

**Table 2.** Prediction models for risk of medical intensive care unit admission (*N* = 182) for hospitalized patients with sepsis.

			Model 1* (Clinical Variables)		Model 2* (CT-derived Body Composition Parameters)		Model 3* (CT-derived Body Composition Parameters and Clinical Variables)	
Variables	Unadjusted Odds Ratio (95% CI)	*p* value	Adjusted Odds Ratio (95% CI)	*p* value	Adjusted Odds Ratio (95% CI)	*p* value	Adjusted Odds Ratio (95% CI)	*p* value
Male sex	0.883 (0.540–1.442)	0.618	**–**	**–**	**–**	**–**	**–**	**–**
Age	1.003 (0.985–1.022)	0.713	**–**	**–**		**–**	0.977 (0.952–1.002)	0.071
BMI	0.952 (0.901–1.006)	0.082	**–**	**–**		**–**	**–**	**–**
COPD	1.981 (0.811–4.840)	0.134	2.148 (0.733–6.296)	0.164	–	–	1.824 (0.605–5.496)	0.286
Heart failure	3.093 (1.788–5.352)	**0.000**	1.741 (0.891–3.404)	0.105	–	–	–	–
Hypertension	1.054 (0.659–1.688)	0.825	–	–	–	–	–	–
CKD	2.597 (1.484–4.545)	**0.001**	–	–	–	–	–	–
Diabetes	0.939 (0.569–1.550)	0.806	–	–	–	–	–	–
Chronic liver disease	5.019 (1.709–14.739)	**0.003**	–	–	–	–	–	–
PaO_2_/FiO_2_	0.988 (0.985–0.992)	**0.000**	0.990 (0.985–0.994)	**0.000**	–	–	0.988 (0.983–0.993)	**0.000**
WBC	1.106 (1.052–1.163)	**0.000**	1.120 (1.045–1.199)	**0.001**	–	–	1.128 (1.051–1.210)	**0.001**
Neutrophil count	1.151 (1.085–1.222)	**0.000**	–	–	–	–	–	–
Lymphocyte count	0.337 (0.219–0.520)	**0.000**	0.366 (0.221–0.605)	**0.000**	–	–	0.365 (0.211–0.632)	**0.000**
Hgb	0.981 (0.971–0.991)	**0.000**	–	–	–	–	–	–
PLT	0.998 (0.996–1.000)	0.127	–	–	–	–	–	–
D dimer	1.892 (1.448–2.474)	**0.000**	1.554 (1.175–2.056)	**0.002**	–	–	1.507 (1.123–2.021)	**0.006**
TNI	0.981 (0.908–1.060)	0.628	–	–	–	–	–	–
ALT	1.006 (1.000–1.012)	0.061	–	–	–	–	–	–
AST	1.012 (1.003–1.021)	**0.010**	–	–	–	–	1.010 (1.000–1.019)	0.053
GGT	1.000 (0.997–1.003)	0.985	–	–	–	–	–	–
ALB	0.892 (0.852–0.934)	**0.000**	0.916 (0.862–0.973)	**0.004**	–	–	0.950 (0.889–1.017)	0.138
Cr	1.004 (1.000–1.007)	0.050	**–**	**–**	**–**	**–**	**–**	**–**
CRP	1.006 (1.003–1.009)	**0.000**	**–**	**–**	**–**	**–**	**–**	**–**
SMA_T4_^a^	0.905 (0.854–0.959)	**0.001**	**–**	**–**	0.801 (0.738–0.870)	**0.000**	0.795 (0.708–0.893)	**0.000**
SMI_T4_^a^	0.693 (0.574–0.836)	**0.000**	**–**	**–**	**–**	**–**	**–**	**–**
SMA_L1_^a^	0.994 (0.922–1.071)	0.868	**–**	**–**	**–**	**–**	**–**	**–**
SMI_L1_^a^	0.969 (0.771–1.219)	0.789	**–**	**–**	**–**	**–**	**–**	**–**
SAT_L1_^a^	0.992 (0.956–1.030)	0.690	**–**	**–**	0.920 (0.871–0.973)	**0.003**	0.897 (0.829–0.971)	**0.007**
AC_L1_^a^	1.165 (0.950–1.430)	0.143	**–**	**–**	2.329 (1.618–3.353)	**0.000**	2.095 (1.271–3.453)	**0.004**
IMAT_L1_^b^	1.032 (0.978–1.088)	0.255	**–**	**–**	–	–	1.060 (0.977–1.149)	0.161

Bold face indicates a *p* value with statistical significance. CT: computed tomography; CI: confidence interval; BMI: body mass index; COPD: chronic obstructive pulmonary disease; CKD: chronic kidney disease; PaO_2:_ arterial partial pressure of oxygen; FiO_2:_ fractional oxygen concentration; WBC: white blood cell; Hgb: hemoglobin; PLT: platelet; TNI: troponin I; ALT: alanine aminotransferase; AST: aspartate transaminase; GGT: gamma-glutamyl transferase; ALB: albumin; Cr: creatinine; CRP: C-reactive protein; SMA_T4:_ skeletal muscle area at T4 level; SMI_T4_: skeletal muscle index at T4 level; SMA_L1_: skeletal muscle area at L1 level; SMI_L1_: skeletal muscle index at L1 level; SAT_L1_: subcutaneous adipose tissue at L1 level; AC_L1_: abdominal circumference at L1 level; IMAT_L1_: intramuscular adipose tissue at L1 level.

* Variables selected through multivariable linear regression with backward elimination (Supplementary Tables 5–7).

^a^ Per 10 units increments.

^b^ Per 1 unit increment.

**Table 3.** Prediction models for risk of death (*N* = 83) for hospitalized patients with sepsis.

			Model 1* (Clinical Variables)		Model 2* (CT-derived Body Composition Parameters)		Model 3* (CT-derived Body Composition Parameters and Clinical Variables)	
Variables	Unadjusted Odds Ratio (95% CI)	*p* value	Adjusted Odds Ratio (95% CI)	*p* value	Adjusted Odds Ratio (95% CI)	*p* value	Adjusted Odds Ratio (95% CI)	*p* value
Male sex	0.916 (0.578–1.452)	0.709	**–**	**–**	**–**	**–**	**–**	**–**
Age	1.025 (1.005–1.046)	**0.016**	–	–	1.022 (1.001–1.044)	**0.041**	–	–
BMI	0.974 (0.917–1.036)	0.405	**–**	–	0.928 (0.849–1.013)	0.095	–	–
COPD	0.706 (0.285–1.747)	0.451	–	–	–	–	–	–
Heart failure	2.395 (1.534–3.737)	**0.000**	1.914 (1.177–3.112)	**0.009**	–	–	3.355 (1.796–6.266)	**0.000**
Hypertension	1.098 (0.702–1.717)	0.682	–	–	–	–	–	–
CKD	1.872 (1.484–4.545)	**0.001**	1.014 (0.604–1.702)	0.958	–	–	3.504 (1.852–6.629)	**0.000**
Diabetes	1.036 (0.647–1.659)	0.883	–	–	–	–	–	–
Chronic liver disease	1.025 (0.576–1.825)	0.932	–	–	–	–	–	–
PaO_2_/FiO_2_	0.993 (0.990–0.995)	**0.000**	0.995 (0.992–0.998)	**0.002**	–	–	0.990 (0.986–0.994)	**0.000**
WBC	1.021 (0.991–1.051)	0.175	–	–	–	–	–	–
Neutrophil count	1.026 (0.995–1.057)	0.102	–	–	–	–	–	–
Lymphocyte count	0.575 (0.354–0.935)	**0.026**	–	–	–	–	–	–
Hgb	0.991 (0.983–1.000)	0.061	–	–	–	–	–	–
PLT	0.999 (0.998–1.001)	0.433	1.001 (1.000–1.003)	0.115	–	–	–	–
D dimer	1.034 (1.008–1.060)	**0.010**	–	–	–	–	–	–
TNI	1.017 (0.949–1.090)	0.631	–	–	–	–	–	–
ALT	1.001 (1.000–1.003)	0.175	–	–	–	–	–	–
AST	1.001 (1.000–1.001)	0.248	–	–	–	–	–	–
GGT	1.000 (0.997–1.002)	0.774	–	–	–	–	–	–
ALB	0.977 (0.938–1.017)	0.247	–	–	–	–	–	–
Cr	1.001 (1.000–1.003)	0.158	**–**	**–**	**–**	**–**	**–**	**–**
CRP	1.002 (0.999–1.004)	0.223	**–**	**–**	**–**	**–**	**–**	**–**
SMA_T4_^a^	0.964 (0.909–1.022)	0.216	**–**	**–**	0.951 (0.887–1.019)	0.154	0.880 (0.800–0.967)	**0.008**
SMI_T4_^a^	0.990 (0.973–1.008)	0.294	**–**	**–**	**–**	**–**	**–**	**–**
SMA_L1_^a^	0.986 (0.916–1.060)	0.700	**–**	**–**	**–**	**–**	**–**	**–**
SMI_L1_^a^	0.999 (0.977–1.021)	0.904	**–**	**–**	**–**	**–**	**–**	**–**
SAT_L1_^a^	0.997 (0.961–1.036)	0.890	**–**	**–**	–	–	–	–
AC_L1_^a^	1.140 (0.936–1.389)	0.192	**–**	**–**	1.501 (1.137–1.982)	**0.004**	1.527 (1.122–2.079)	**0.007**
IMAT_L1_^b^	1.020 (0.987–1.055)	0.239	**–**	**–**	–	–	–	–

Bold face indicates a *p* value with statistical significance. CT: computed tomography; CI: confidence interval; BMI: body mass index; COPD: chronic obstructive pulmonary disease; CKD: chronic kidney disease; PaO_2:_ arterial partial pressure of oxygen; FiO_2:_ fractional oxygen concentration; WBC: white blood cell; Hgb: hemoglobin; PLT: platelet; TNI: troponin I; ALT: alanine aminotransferase; AST: aspartate transaminase; GGT: gamma-glutamyl transferase; ALB: albumin; Cr: creatinine; CRP: C–reactive protein; SMA_T4_: skeletal muscle area at T4 level; SMI_T4_: skeletal muscle index at T4 level; SMA_L1_: skeletal muscle area at L1 level; SMI_L1_: skeletal muscle index at L1 level; SAT_L1:_ subcutaneous adipose tissue at L1 level; AC_L1:_ abdominal circumference at L1 level; IMAT_L1_: intramuscular adipose tissue at L1 level.

* Variables selected through multivariable linear regression with backward elimination (Supplementary Tables 5–7).

^a^ Per 10 units increments.

^b^ Per 1 unit increment.

In the multivariate logistic regression analysis of Model 3 (which included clinical variables and CT-based body composition parameters), high AC_L1_ remained significantly associated with an increased risk of MICU admission (OR 2.095, 95% CI 1.271–3.453, *p* = 0.004; Table 2) and was a significant risk factor for septic shock (OR 1.485, 95% CI 1.054–2.094, *p* = 0.024; Supplementary Table 4) as well as in-hospital mortality (OR 1.527, 95% CI 1.122–2.079, *p* = 0.007; Table 3). Additionally, when assessing the risk of in-hospital mortality in Model 3, high SMA_T4_ was independently associated with reduced mortality (OR 0.880, 95% CI 0.800–0.967, *p* = 0.008; Table 3), and this association persisted after adjusting for the PaO_2_/FiO_2_ ratio, heart failure, and CKD.

### ROC comparison

**Figure 3.**
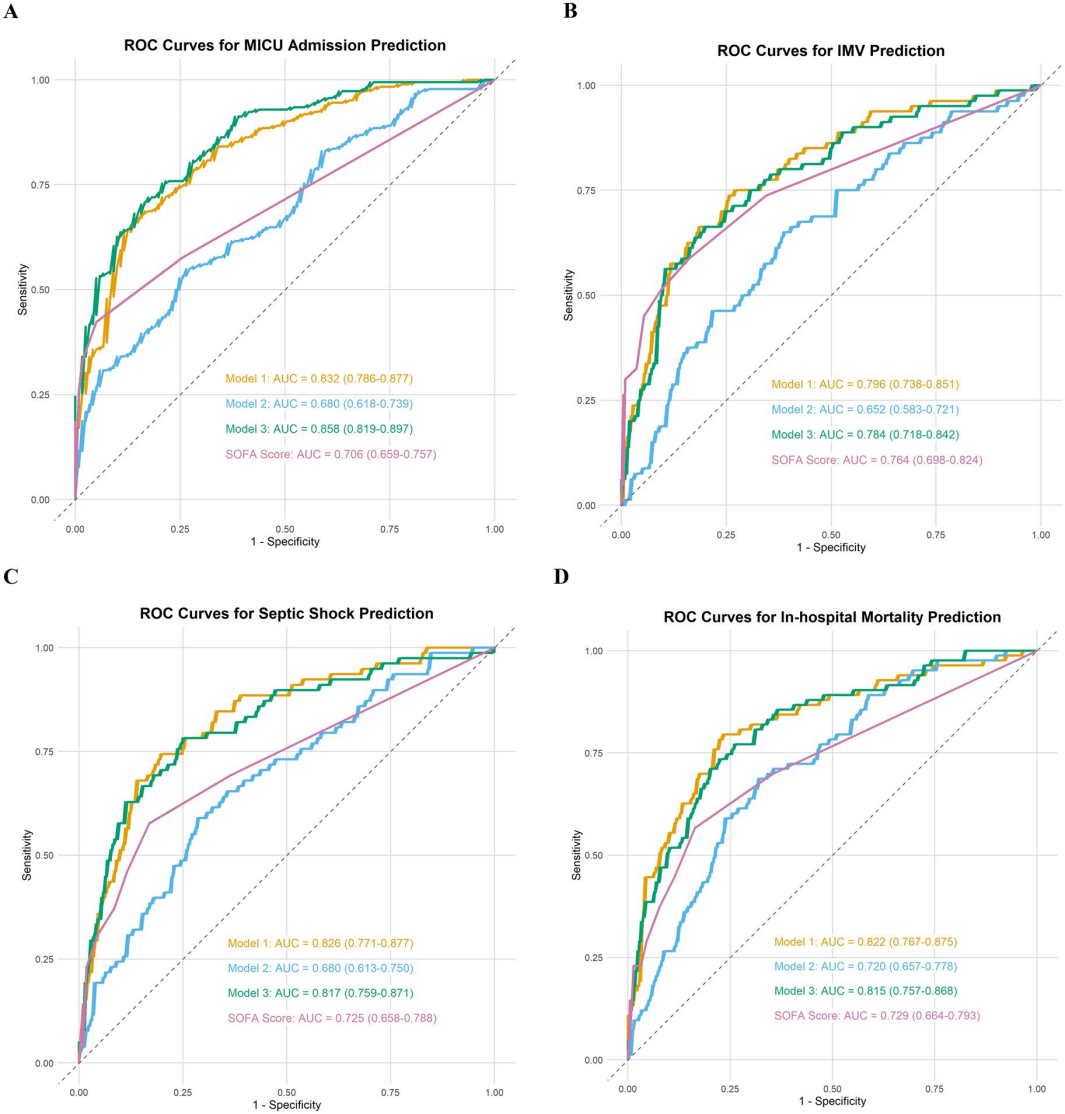
Receiver operating characteristic curve analyses of the obtained models and SOFA score for the outcome prediction. (A) Medical intensive care unit (MICU) admission prediction: Model 1 incorporated only clinical variables, including COPD, heart failure, PaO_2_/FiO_2_ ratio, WBC count, lymphocyte count, ALB, and D-dimer, Model 2 incorporated only body composition parameters, including SMA_T4_, SAT_L1_, and AC_L1_, and Model 3 incorporated both clinical variables and body composition parameters, including age, COPD, PaO_2_/FiO_2_ ratio, WBC count, AST, ALB, lymphocyte count, D-dimer, SMA_T4_, SAT_L1_, IMAT_L1_, and AC_L1_; (B) Invasive mechanical ventilation (IMV) prediction: Model 1 incorporated only clinical variables, including PaO_2_/FiO_2_ ratio, AST, D-dimer, CKD, and neutrophil count, Model 2 incorporated only body composition parameters, including age, SMI_T4_, and AC_L1_, and Model 3 incorporated both clinical variables and body composition parameters, including PaO_2_/FiO_2_ ratio, AST, CKD, neutrophil count, and AC_L1_; (C) Septic shock prediction: Model 1 incorporated only clinical variables, including heart failure, CKD, PaO_2_/FiO_2_ ratio, Hgb, and AST, Model 2 incorporated only body composition parameters, including BMI, SMI_T4_, SMA_L1_, and AC_L1_, and Model 3 incorporated both clinical variables and body composition parameters, including PaO_2_/FiO_2_ ratio, CKD, AST, AC_L1_, and SAT_L1_; (D) In-hospital mortality prediction: Model 1 incorporated only clinical variables, including age, PaO_2_/FiO_2_ ratio, CKD, heart failure, and AST, Model 2 incorporated only body composition parameters, including age, BMI, AC_L1_, SMA_T4_, and Model 3 incorporated both clinical variables and body composition parameters, including PaO_2_/FiO_2_ ratio, CKD, heart failure, SMA_T4_, AC_L1_. ROC: receiver operating characteristic; AUC: area under the curve; SOFA: sequential organ failure assessment.

For predicting MICU admission, Model 1 had a five-fold cross-validated average AUC of 0.842 and an overall AUC of 0.832. Model 2 had a five-fold cross-validated average AUC of 0.681 and an overall AUC of 0.680. Model 3 achieved a five-fold cross-validated average AUC of 0.865 and an overall AUC of 0.858 (Figure 3A and Supplementary Figure 5A–C). There was a significant difference between the overall AUCs of Model 1 and Model 3 (AUC 0.832 versus AUC 0.858, *p* = 0.047), and Model 3 achieved the highest sensitivity of 0.703 (Supplementary Table 8). Similarly, regarding the prediction of IMV, septic shock, and in-hospital mortality, both the average and overall AUC values for each model demonstrated a high degree of consistency in performance (Figure 3B–D and Supplementary Figure 5D–L). There was no statistically difference was found between Model 1 and Model 3 for predicting IMV (AUC 0.796 versus AUC 0.784, *p* = 0.139), septic shock (AUC 0.826 versus AUC 0.817, *p* = 0.442), and in-hospital mortality (AUC 0.822 versus AUC 0.815, *p* = 0.703) (Supplementary Table 8). Although Model 2 predicted IMV significantly worse than SOFA scores (AUC 0.652 versus AUC 0.764, *p* = 0.022), the predictive accuracy for MICU admission (AUC 0.680 versus AUC 0.706, *p* = 0.493), septic shock (AUC 0.680 versus AUC 0.725, *p* = 0.361), and in-hospital mortality (AUC 0.720 versus AUC 0.729, *p* = 0.840) were comparable to SOFA scores (Supplementary Table 8).

### Body composition parameter stratification

**Figure 4.**
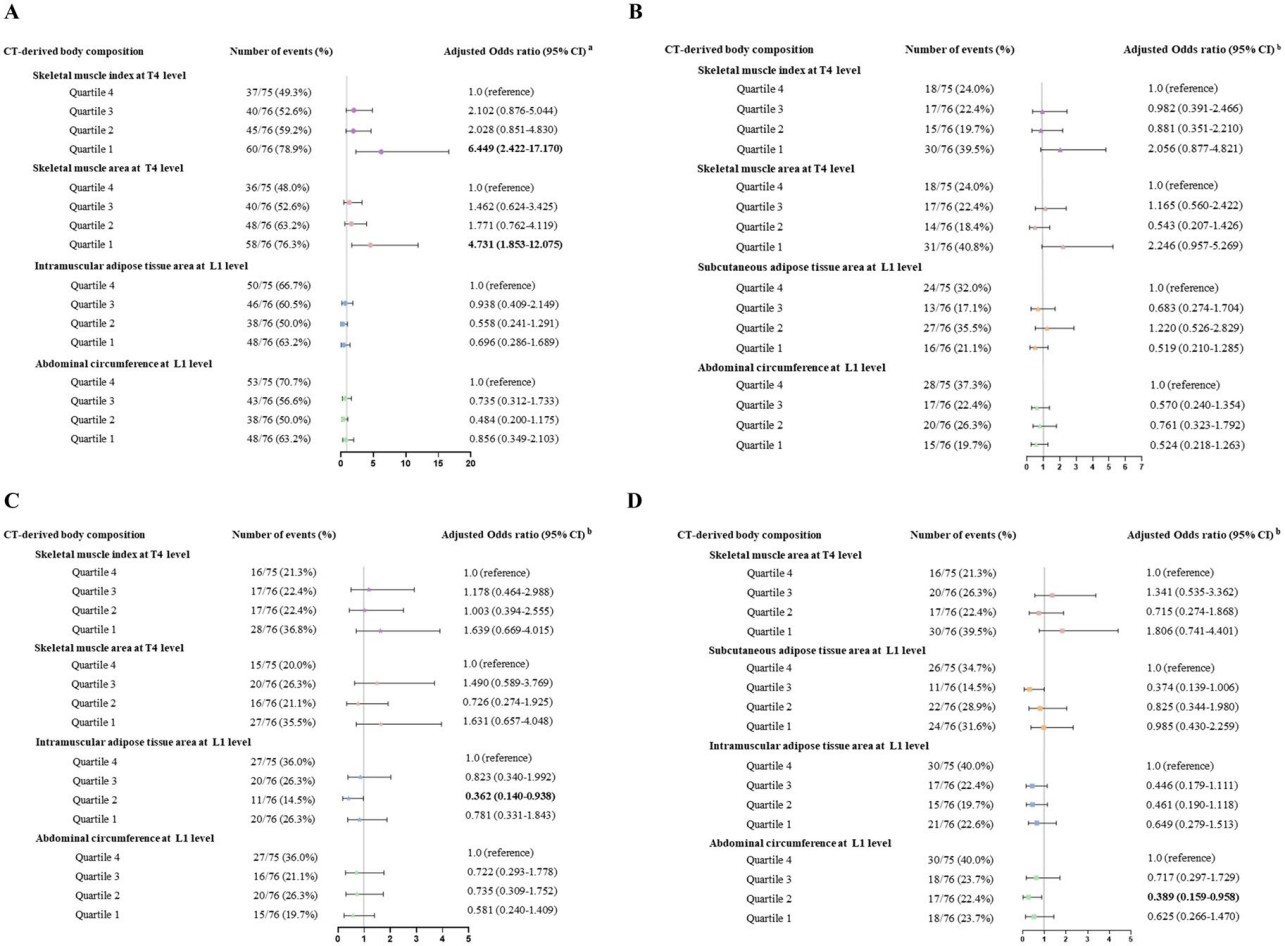
Forest plots of the adjusted odds ratio of adverse events including (A) medical intensive care unit admission, (B) invasive mechanical ventilation, (C) septic shock, and (D) mortality in multivariate binary logistic regression analyses. Forest plots according to sex-specific quartiles show the association between computed tomography (CT)-derived body composition parameters and adverse events. Bold face indicates a *p* value with statistical significance. Colered geometry indicate odds ratio; error bar, 95% confidence interval (CI). ^a^After adjusting to SOFA score, PaO_2_/FiO_2_, CKD, heart failure, chronic liver disease, WBC, Hgb, ALB, D dimer, AST, and CRP. ^b^After adjusting to SOFA score, PaO_2_/FiO_2_, CKD, and heart failure.

Compared with patients with SMI_T4_ or SMA_T4_ in the fourth quartile, patients in the first quartile had the highest odds of requiring admission to MICU, IMV, septic shock, and mortality (Supplementary Figure 4A–D). These patients remained at high risk of admission to MICU after multivariable adjustment for SOFA score, PaO_2_/FiO_2_ ratio, CKD, heart failure, chronic liver disease, WBC, Hgb, ALB, D-dimer, AST, and C-reactive protein (CRP) (SMA_T4_: OR 4.731, 95% CI 1.853–12.075; SMI_T4_: OR 6.449, 95% CI 2.422–17.170) (Figure 4A). In terms of fat mass, we found that only patients in the second quartile of IMAT_L1_ exhibited a notably lower risk of developing septic shock compared to those in the fourth quartile, independent of SOFA score, PaO_2_/FiO_2_ ratio, CKD, and heart failure (OR 0.362, 95% CI 0.140–0.938, *p* = 0.036) (Figure 4C). Additionally, only patients in the second quartile of AC_L1_ exhibited a lower risk of mortality (OR 0.389, 95% CI 0.158–0.958, *p* = 0.040), not MICU admission, IMV, or septic shock, compared to those in the fourth quartile after the similar adjustments (Figure 4A–D).

## Discussion

There are several important findings in this retrospective study. Firstly, high thoracic muscle area but not abdominal muscle area, high SAT_L1_ and low AC_L1_ are independent protective factors against admitted to the MICU in sepsis patients with pneumonia. Low thoracic muscle area and high AC_L1_ are also independently associated with in-hospital mortality. Secondly, high IMAT_L1_ was identified as an independent risk factor for the decline in the PaO_2_/FiO_2_ ratio during hospitalization. Thirdly, the integration of CT-derived body composition parameters with conventional clinical risk factors enhanced the predictive capability for MICU admission in patients with sepsis. The model developed utilizing CT-derived body composition parameters demonstrated a performance in predicting adverse events including MICU admission, septic shock, and mortality in patients with sepsis that was comparable to the SOFA scores.

A growing body of research has substantiated the role of CT-derived muscle CSA in prognosticating outcomes for critically ill patients. For instance, Koehler et al. [25] identified the T4 muscle CSA as an independent risk factor for ICU admission in the context of SARS-CoV-2 infection. Similarly, Van Bakel et al. [12] reported an association between the T4 pectoralis muscle CSA and 30-day in-hospital mortality among patients with COVID-19. Furthermore, Darden et al. [26] demonstrated that reduced muscle mass at the L3 level independently predicted one-year mortality in patients with sepsis. Unlike these studies, our study focuses on the total muscle CSA assessment at the T4 and L1 levels for sepsis patients with pneumonia. The clinical significance is as follows: (1) Sepsis with pneumonia is the most common subtype of sepsis [2], and managing its prognosis holds significant importance for public health; (2) Chest CT is a routine method for diagnosing pneumonia-associated sepsis, and using it to assess body composition offers natural accessibility and convenience, without the need for additional examinations; (3) Multiple thoracic muscle groups, such as the pectoralis major, intercostal muscles, latissimus dorsi, and erector spinae are involved in respiratory function [27], making the assessment of T4 muscle CSA particularly relevant from a pathophysiological standpoint and potentially providing stronger predictive value. Our results support this view, that is, SMA_T4_, rather than SMA_L1_, is a powerful indicator for predicting the admission to the MICU and the death of sepsis patients with pneumonia. Additionally, patients in the lowest quartile of SMI_T4_, which represents severe muscle atrophy, had the highest risk of admission to the MICU, independent of SOFA score, comorbidities, and other clinical risk factors. This suggests that the loss of thoracic muscle mass detected by chest CT can serve as an imaging biomarker for the potential frailty status [28], which might impair the patients’ ability to cope with the attack of sepsis, thereby leading to worse prognosis. Several possible explanations are: Firstly, under the dual pressure of acute respiratory failure caused by pneumonia and the high metabolic demands of sepsis, this deficiency in muscle reserve may lead to earlier or more severe respiratory failure, increasing the need for admission to the MICU and the risk of death. Secondly, low muscle mass is associated with chronic low-grade inflammatory states and immune dysfunction [29]. In the context of the acute inflammatory storm of sepsis, low muscle mass may amplify the inflammatory response, exacerbate organ damage, and weaken the ability to fight infection, ultimately leading to a worse prognosis [30]. Thirdly, skeletal muscle is an important metabolic organ and a reservoir of proteins [31]. A reduction in muscle mass indicates a decline in metabolic reserves [32]. In the hypercatabolic state of sepsis, this decline may lead to a faster depletion of these reserves, making it difficult to maintain the necessary conditions for organ function repair and, thus, affecting recovery. In a word, the low SMAT4 detected by CT is not merely an imaging finding; it is also a quantitative reflection of the patient’s physiological fragility, suggesting a significant reduction in their ability to withstand the impact of sepsis.

The present study found that high AC_L1_, serving as a surrogate measure of visceral adiposity, was as an independent risk factor for MICU admission, the onset of septic shock, and mortality in sepsis patients with pneumonia. This may be due to the fact that visceral fat, as an ‘endocrine organ’, releases a large amount of free fatty acids (FFA), pro-inflammatory cytokines (such as interleukin-6, tumour necrosis factor-α, and leptin), and pro-thrombotic factors, amplifying the already existing inflammatory response in sepsis, inducing insulin resistance and lipid metabolism disorders, and promoting endothelial dysfunction and a hypercoagulable state [33–35]. These extensive metabolic inflammatory disorders constitute the core of the patient’s inherent vulnerabilities, significantly weakening the reserve of multiple organ function and ultimately driving a higher risk of death. Unlike the ‘systemic attack’ of visceral fat, the increase in IMAT may mainly affects the course of the disease by impairing the local muscle microenvironment and the functions of specific organs [36]. Yang et al. [37] previously demonstrated a significant association between intramuscular fat (IMF) deposition and an increased risk of mechanical ventilation in patients with COVID-19. Our study consistently found that IMAT_L1_ is associated with the deterioration of the oxygenation index during the course of sepsis with pneumonia. The possible mechanism is as follows: local inflammatory factors (such as monocyte chemoattractant protein-1) secreted by intramuscular fat and lipid toxic metabolites directly damage mitochondrial function as well as the contractility and endurance of skeletal muscles (especially the diaphragm), which leads to respiratory muscle weakness, insufficient pulmonary ventilation, and accelerated respiratory failure related to pneumonia [38,39]. Notably, patients with AC_L1_ in the second quartile had a significantly lower mortality risk than those in the highest quartile; likewise, patients with IMAT_L1_ in the second quartile had a significantly lower risk of septic shock than those in the highest quartile. The fact is that fat mass is a risk factor exhibiting a U-shaped relationship [40], indicating that individuals with moderate fat accumulation may have a protective advantage compared to those experiencing cachexia or extreme obesity. Therefore, AC_L1_ and IMAT_L1_ evaluated by CT, may represent different manifestations of metabolic dysregulation-related obesity in terms of spatial distribution (visceral vs. muscle), and together they constitute the imaging biomarkers of ‘metabolic vulnerability’ in patients with sepsis. Unlike AC_L1_ and IMAT_L1_, high SAT_L1_ confers a protective effect against MICU admission and does not have an independent association with the onset of septic shock and death. This might be due to: (1) the inflammatory factor secretion capacity of subcutaneous fat being much lower than that of visceral fat [41]; (2) some studies have shown that subcutaneous fat can secrete anti-inflammatory adipokines (such as adiponectin), but its protective effect in sepsis may be masked by the coexisting visceral fat or IMF deposition [42,43]. In summary, the function of adipose tissue is intricate, with its protective or detrimental impacts being significantly influenced by factors such as its quantity, distribution, metabolic state, and the particular disease context. It is crucial to acknowledge that, while our study found that these body composition parameters derived from chest CT are significantly associated with adverse outcomes, this merely indicates a correlation. The causal relationship and potential biological mechanisms between them need to be further verified through prospective studies.

Although the SOFA score is already part of standard patient assessment in daily practice and has a high predictive ability for in-hospital mortality [17], it relies on extensive laboratory tests and some routine measurements in the ICU that are relatively more difficult and time-consuming to retrieve. It is worth noting that this study found that Model 3 (which includes age, COPD, PaO_2_/FiO_2_ ratio, WBC count, AST, ALB, lymphocyte count, D-dimer, SMA_T4_, SAT_L1_, IMAT_L1_, AC_L1_, with an AUC of 0.858) had higher accuracy in predicting the MICU admission compared to Model 1 (which includes COPD, heart failure, PaO_2_/FiO_2_ ratio, WBC count, lymphocyte count, ALB, D-dimer, with an AUC of 0.832) and the SOFA score (AUC = 0.706). This provides strong evidence that assessing body composition can offer valuable additional information for prognostic assessment based on traditional tools such as the SOFA score, helping to more accurately identify high-risk patients whose conditions may not be fully recognized by conventional parameters. Although the model constructed solely based on CT body composition has a slightly lower predictive ability for IMV compared to the SOFA score, its predictive accuracy for MICU admission, septic shock, and mortality is comparable to that of the SOFA score. These findings indicate that, in situations where conventional clinical and laboratory data have not been fully obtained or analyzed, using readily available chest CT images to quickly assess body composition parameters can serve as an effective supplementary method to provide timely and reliable prognostic information for early risk stratification and intervention decisions, such as prioritizing transfer to the MICU. Notably, our data demonstrated a significant association between body composition parameters and age and sex; however, age- and sex-specific cutoff values of these parameters at different anatomical levels have yet to be established, which require further investigation in large cohorts. Additionally, the integration of artificial intelligence for the fully automated segmentation and analysis of body composition derived from CT scans could significantly reduce the time required to acquire such prognostic information [44].

Several limitations of our study warrant consideration, beyond its single-center and retrospective design. Firstly, the study did not include an analysis of chest CT features (e.g. ground-glass opacities, consolidations, and pleural effusions), which impact the prognosis of sepsis patients with pneumonia. Secondly, it is imperative to consider the potential for selection bias in the inclusion of patients within the study cohort, as individuals lacking chest CT imaging or complete data sets were excluded. Thirdly, the study lacked external validation of the predictive models, which should be prioritized in future multicenter studies. Finally, our study lacked subgroup analyses based on patients’ pathogenic microbes; including these analyses could enhance our model’s predictive accuracy for various pneumonia types.

## Conclusion

The frailty status and visceral obesity, as assessed by chest CT measurements of low thoracic muscle mass and high AC, are independently associated with admission to the MICU and mortality in sepsis patients with pneumonia. The predictive accuracy of the model constructed using CT-derived body composition parameters for adverse events in patients with sepsis is comparable to that of the SOFA score. Chest CT scans provide a valuable supplementary tool for the early risk stratification and management planning of sepsis patients. They can be combined with standard vital sign monitoring and laboratory analysis to achieve a more comprehensive assessment. Future prospective studies should further explore their potential as routine monitoring indicators for optimizing patient risk stratification and individualized management decisions.

## Supplementary Material

Clean copy _Supplementary_Materials - IANN-2025-2746.R1.docx

## Data Availability

The data that support the findings of this study are available from the corresponding author, [R.Z.], upon reasonable request.
