# Supplementary material for: Chest CT-derived body composition parameters for outcome prediction in sepsis patients with pneumonia
Source: Ann Med. 2025 Nov 10;57(1):2584421. doi: 10.1080/07853890.2025.2584421 (PMC12604109; doi:10.1080/07853890.2025.2584421)
Supplement: Clean copy _Supplementary_Materials - IANN-2025-2746.R1.docx [file IANN_A_2584421_SM7051.docx]

**A B C D**

**E F G**

**Supplementary Figure 1. Comparison of CT-derived body composition parameters in each age quartile.** Violin diagram of the median of skeletal muscle area at T4 level (A), skeletal muscle area at L1 level (B), abdominal circumference at L1 level (C), subcutaneous adipose tissue area at L1 level (D), intramuscular adipose tissue area at L1 level (E), skeletal muscle index at T4 level (F), and skeletal muscle index at L1 level (G). CT computed tomography; T4 fourth thoracic vertebra; L1 first lumbar vertebra.

**A B C D**

**Supplementary Figure 2. Sex-related distribution of the median of CT-derived body composition parameters.** (A) Comparison of the skeletal muscle index at T4 and L1 levels between males and females. (B) Comparison of the skeletal muscle area at T4 and L1 levels between males and females. (C) Comparison of the subcutaneous and intramuscular adipose tissue area at L1 level between males and females. (D) Comparison of the abdominal circumference at L1 level between males and females. CT computed tomography; T4 fourth thoracic vertebra; L1 first lumbar vertebra; SMI skeletal muscle index; SMA skeletal muscle area; SAT subcutaneous adipose tissue; IMAT intramuscular adipose tissue; AC abdominal circumference.

**A B**

**Supplementary Figure 3. Correlation between CT-based muscle indexes at L3, L1, and T4 levels (N=126).** (A) The correlation between the muscle index at L3 level and the muscle index at T4 level. (B) The correlation between the muscle index at L3 level and the muscle index at L1 level. CT computed tomography; T4 fourth thoracic vertebra; L1 first lumbar vertebra; L3 third lumbar vertebra; CI confidence interval.

**A B**


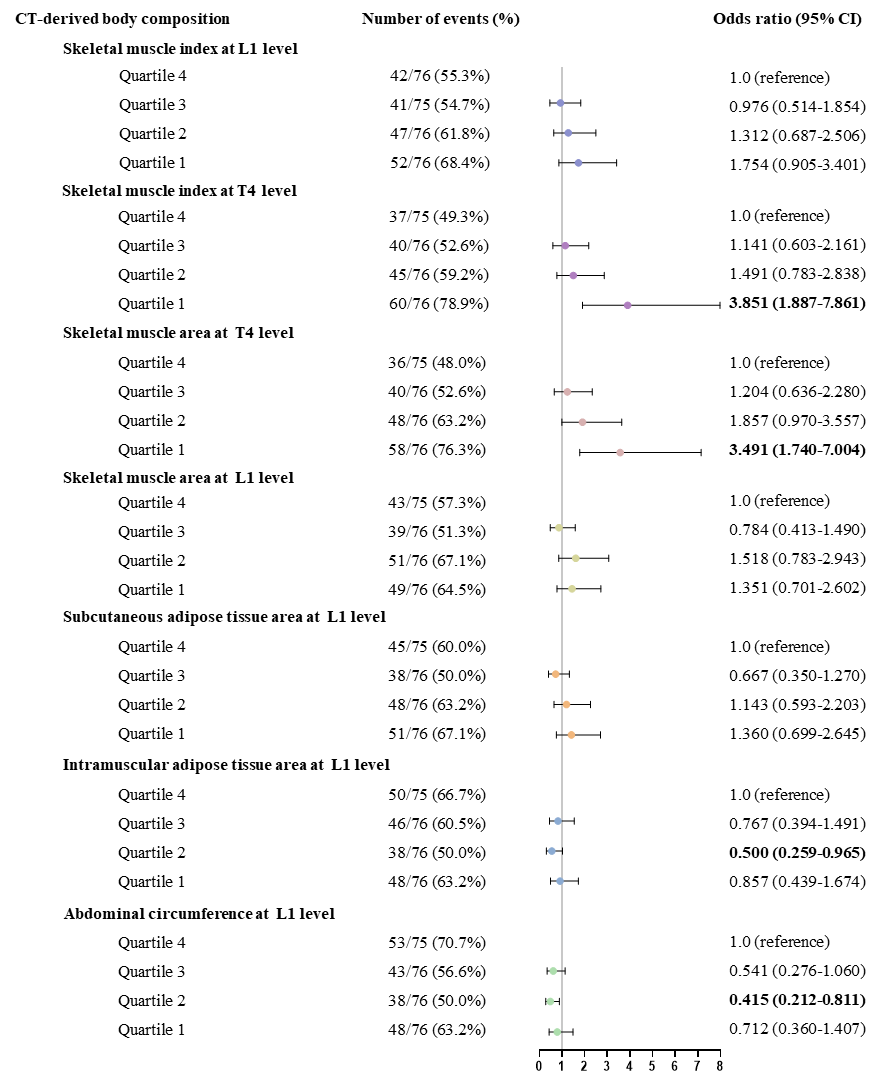

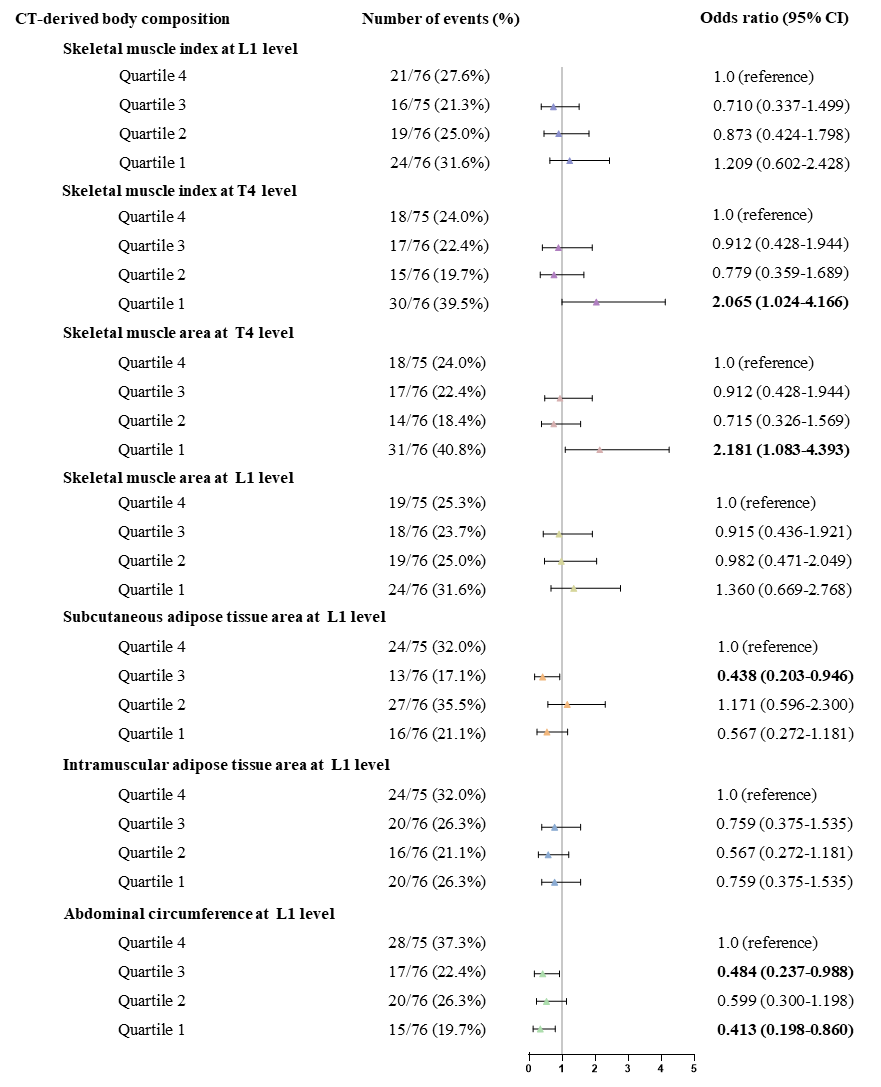


**C D**


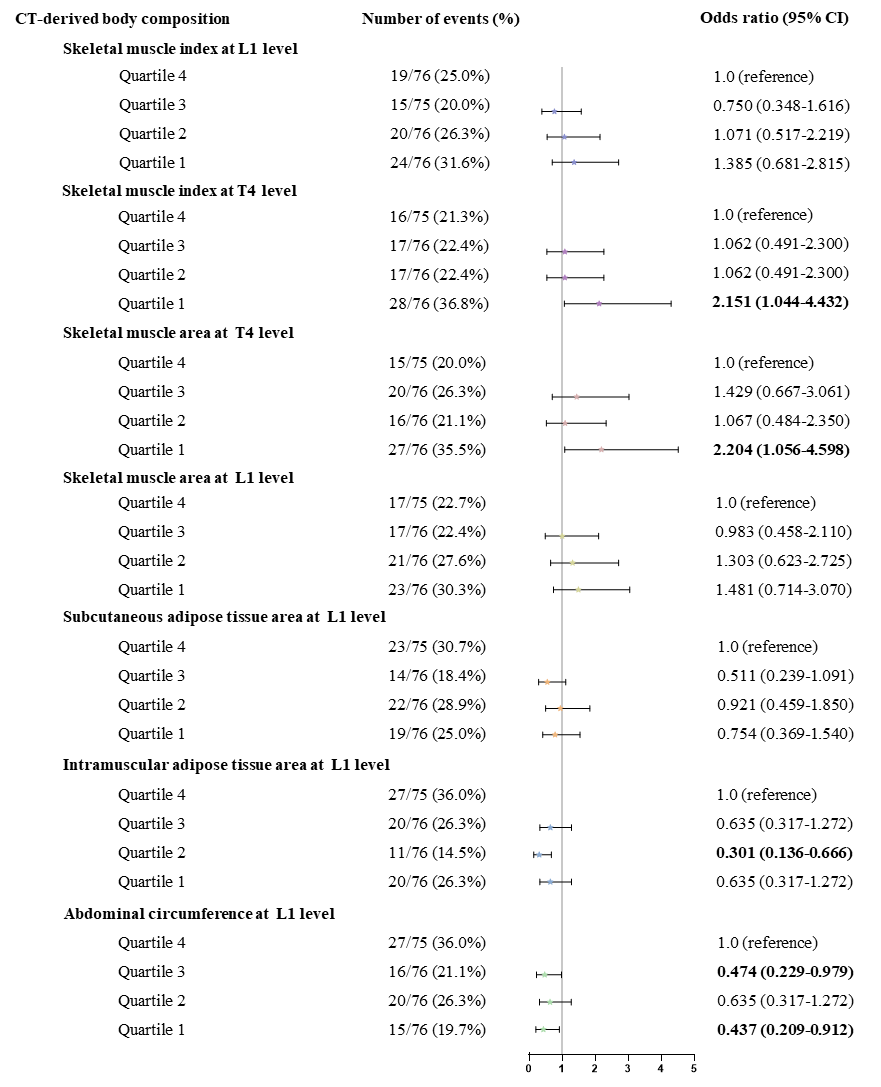
 **
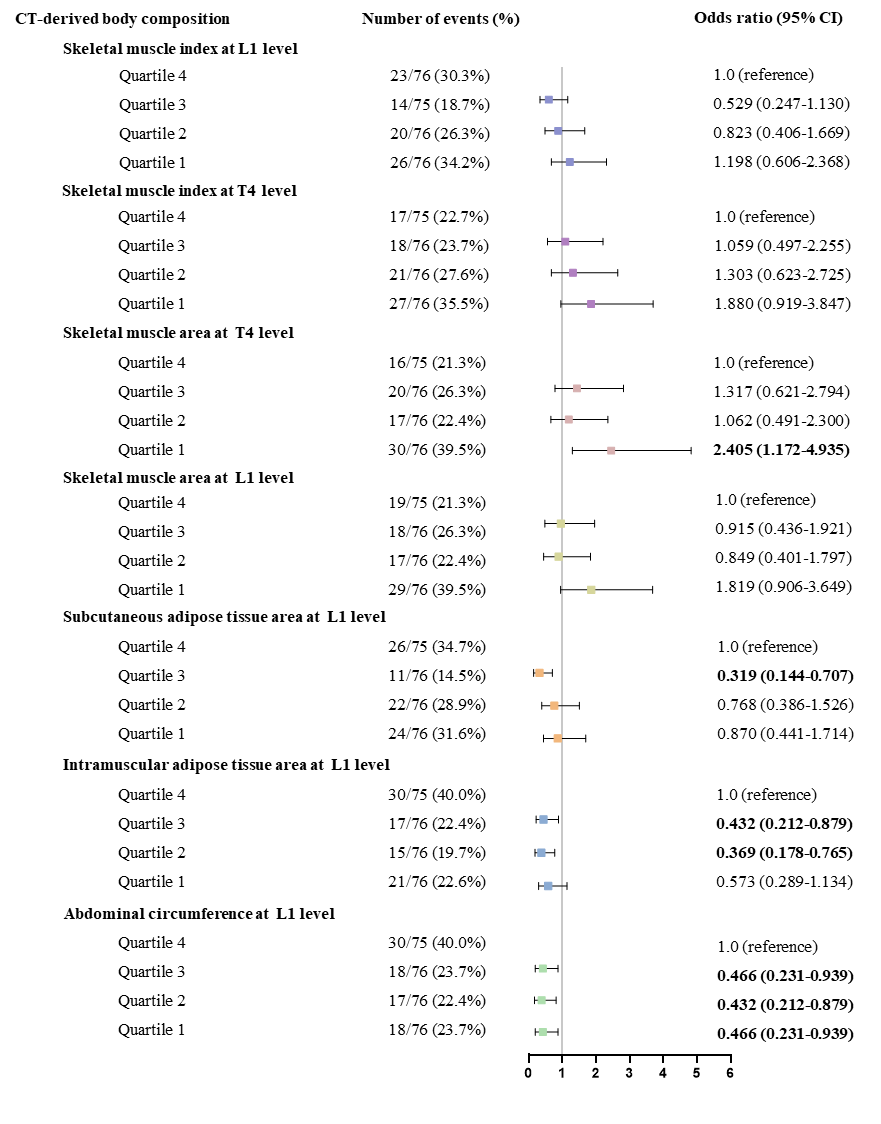
**

**Supplementary Figure 4. Forest plots of the odds ratio of adverse events including (A) medical intensive care unit admission, (B) invasive mechanical ventilation, (C) septic shock, and (D) mortality in univariate binary logistic regression analyses.** Forest plots according to sex-specific quartiles show the association between CT-derived body composition parameters and adverse events. Bold face indicates a *P* value with statistical significance. Colered geometry indicate odds ratio; error bar, 95% CI. CT computed tomography; CI confidence interval.

**Supplementary Table 1. Univariate linear regression analysis of clinical variables and CT-derived body composition parameters for predicting the deterioration of SOFA scores and PaO_2_/FiO_2_ ratio and the length of hospital stay in patients with sepsis**

|  | **△PaO_2_/FiO_2_ ratio ^a^** | |  | **△SOFA score ^b^** | |  | **Hospital length of stay** | |
| --- | --- | --- | --- | --- | --- | --- | --- | --- |
|  | ***β*-coefficient** | ***P* value** |  | ***β*-coefficient  *P* value** | |  | ***β*-coefficient  *P* value** | |
| Male sex | 9.564 | 0.258 |  | -0.079 | 0.848 |  | 0.431 | 0.732 |
| Age | -0.394 | 0.221 |  | 0.047 | **0.003** |  | 0.049 | 0.310 |
| BMI | 0.692 | 0.461 |  | -0.064 | 0.164 |  | -0.111 | 0.428 |
| COPD | -0.087 | 0.995 |  | 0.182 | 0.790 |  | 0.101 | 0.961 |
| Heart failure | -28.442 | **0.001** |  | 2.647 | **0.007** |  | 2.127 | 0.094 |
| Hypertension | -2.537 | 0.755 |  | -0.066 | 0.869 |  | 0.557 | 0.646 |
| CKD | -9.740 | 0.270 |  | 2.178 | **0.000** |  | 4.037 | **0.002** |
| Diabetes | -6.547 | 0.452 |  | 0.045 | 0.915 |  | 0.750 | 0.563 |
| Chronic liver disease | -7.325 | 0.578 |  | 2.058 | **0.001** |  | 8.631 | **0.000** |
| SOFA score | -0.891 | 0.595 |  | 0.235 | **0.004** |  | 0.399 | 0.109 |
| PaO_2_/FiO_2_ | -0.107 | **0.034** |  | -0.015 | **0.000** |  | -0.009 | 0.255 |
| WBC | -1.059 | 0.087 |  | 0.092 | **0.002** |  | 0.154 | 0.094 |
| Neutrophil count | -1.374 | **0.036** |  | 0.111 | **0.001** |  | 0.211 | **0.030** |
| Lymphocyte count | 18.366 | **0.004** |  | -0.776 | **0.012** |  | -1.913 | **0.042** |
| Hgb | 0.277 | 0.087 |  | -0.026 | **0.001** |  | -0.047 | 0.053 |
| PLT | -0.070 | 0.057 |  | 0.001 | 0.590 |  | 0.007 | 0.192 |
| D dimer | 0.003 | 0.997 |  | 0.038 | 0.321 |  | 0.080 | 0.495 |
| TNI | 0.618 | 0.646 |  | -0.018 | 0.783 |  | 0.108 | 0.588 |
| ALT | -0.054 | 0.275 |  | 0.005 | **0.038** |  | 0.009 | 0.241 |
| AST | -0.058 | 0.074 |  | 0.004 | **0.007** |  | 0.010 | **0.043** |
| GGT | -0.014 | 0.790 |  | 0.001 | 0.662 |  | 0.236 | **0.009** |
| ALB | 0.878 | 0.212 |  | -0.028 | 0.411 |  | -0.175 | 0.094 |
| Cr | -0.032 | 0.450 |  | 0.002 | 0.317 |  | 0.006 | 0.355 |
| CRP | -0.076 | 0.109 |  | 0.004 | 0.081 |  | 0.001 | 0.870 |
| SMA_T4_ ^c^ | 2.222 | **0.021** |  | -0.105 | **0.001** |  | -0.209 | 0.146 |
| SMI_T4_ ^c^ | 7.851 | **0.010** |  | -0.499 | **0.001** |  | -0.668 | 0.142 |
| SMA_L1_ ^c^ | 1.632 | 0.208 |  | -0.103 | 0.106 |  | 0.074 | 0.701 |
| SMI_L1_ ^c^ | 5.379 | 0.176 |  | -0.287 | 0.140 |  | 0.252 | 0.671 |
| SAT_L1_ ^c^ | -0.647 | 0.324 |  | 0.102 | 0.707 |  | 0.071 | 0.469 |
| AC_L1_ ^c^ | -1.021 | 0.769 |  | 0.252 | 0.139 |  | 0.722 | 0.163 |
| IMAT_L1_ ^d^ | -2.384 | **0.004** |  | 0.079 | 0.055 |  | 0.054 | 0.668 |

Bold face indicates a *P* value with statistical significance. CT computed tomography; BMI body mass index; COPD chronic obstructive pulmonary disease; CKD chronic kidney disease; PaO_2_ arterial partial pressure of oxygen; FiO_2_ fractional oxygen concentration; SOFA sequential organ failure assessment; WBC white blood cell; Hgb hemoglobin; PLT platelet; TNI troponin I; ALT alanine aminotransferase; AST aspartate transaminase; GGT gamma-glutamyl transferase; ALB albumin; Cr creatinine; CRP C-reactive protein; SMA_T4_ skeletal muscle area at T4 level; SMI_T4_ skeletal muscle index at T4 level; SMA_L1_ skeletal muscle area at L1 level; SMI_L1_ skeletal muscle index at L1 level; SAT_L1_ subcutaneous adipose tissue at L1 level; AC_L1_ abdomen circumference at L1 level; IMAT_L1_ intramuscular adipose tissue at L1 level.

^c^ Per 10 units increments; ^d^ Per 1 unit increment.

^a^△PaO_2_/FiO_2_ = Lowest PaO_2_/FiO_2_ - PaO_2_/FiO_2_ at admission

^b^△SOFA score = Highest SOFA score - SOFA score at admission

**Supplementary Table 2. Multivariate linear regression analysis of clinical variables and CT-derived body composition parameters for predicting the deterioration of SOFA scores and PaO_2_/FiO_2_ ratio and the length of hospital stay in patients with sepsis**

|  | **△PaO_2_/FiO_2_ ^a^** | | | **△SOFA score ^b^** | | | **Hospital length of stay** | | |
| --- | --- | --- | --- | --- | --- | --- | --- | --- | --- |
|  | ***β*-coefficient** | **95%CI** | ***P* value** | ***β*-coefficient** | **95%CI** | ***P* value** | ***β*-coefficient** | **95%CI** | ***P* value** |
| **Independent variable** |  |  |  |  |  |  |  |  |  |
| Age | - | - | - | 0.019 | -0.010, 0.049 | 0.195 | **-** | **-** | **-** |
| Heart failure | -23.114 | -39.739, -6.490 | **0.007** | 1.511 | 0.727, 2.296 | **0.000** | **-** | **-** | **-** |
| CKD | - | - | - | 1.455 | 0.631, 2.278 | **0.001** | 2.160 | -0.469, 4.789 | 0.107 |
| Chronic liver disease | - | - | - | 0.206 | -0.985, 1.398 | 0.733 | 6.807 | 2.913, 10.701 | **0.001** |
| PaO_2_/FiO_2_ | -0.197 | -0.295, -0.099 | **0.000** | -0.011 | -0.016, -0.007 | **0.000** | - | - | - |
| SOFA score |  |  |  | -0.163 | -0.333, 0.007 | 0.061 |  |  |  |
| WBC | - | - | - |  |  |  | - | - | - |
| Neutrophil count | -1.467 | -2.718, -0.216 | **0.022** | 0.046 | -0.013, 0.106 | 0.126 | 0.119 | -0.071, 0.309 | 0.219 |
| Lymphocyte count | 16.346 | 4.309, 28.383 | **0.008** | -0.191 | -0.741, 0.359 | 0.495 | -1.445 | -3.248, 0.357 | 0.116 |
| Hgb | - | - | - | -0.010 | -0.025, 0.006 | 0.230 | - | - | - |
| ALT | - | - | - | 0.000 | -0.008, 0.007 | 0.910 | - | - | - |
| AST | - | - | - | 0.003 | -0.002, 0.008 | 0.216 | 0.006 | -0.003, 0.015 | 0.203 |
| GGT | **-** | **-** | **-** | - | - | - | 0.005 | -0.010, 0.020 | 0.507 |
| SMA_T4_ ^c^ | -0.083 | -5.160, 4.995 | 0.974 | -0.023 | -0.261, 0.216 | 0.852 | **-** | **-** | **-** |
| SMI_T4_ ^c^ | 8.016 | -8.225, 24.257 | 0.332 | -0.147 | -0.886, 0.592 | 0.696 | **-** | **-** | **-** |
| IMAT_L1_ ^d^ | -2.360 | -3.928, -0.791 | **0.003** | - | - | - | **-** | **-** | **-** |
| Durbin-Waston statistic | 1.744 | | | 1.555 | | | 1.866 | | |

Bold face indicates a *P* value with statistical significance. CT computed tomography; CKD chronic kidney disease; PaO_2_ arterial partial pressure of oxygen; FiO_2_ fractional oxygen concentration; SOFA sequential organ failure assessment; WBC white blood cell; Hgb hemoglobin; ALT alanine aminotransferase; AST aspartate transaminase; GGT gamma-glutamyl transferase; SMA_T4_ skeletal muscle area at T4 level; SMI_T4_ skeletal muscle index at T4 level; IMAT_L1_ intramuscular adipose tissue at L1 level.

^a^△PaO_2_/FiO_2_ = Lowest PaO_2_/FiO_2_ - PaO_2_/FiO_2_ at admission

^b^△SOFA score = Highest SOFA score - SOFA score at admission

^c^ Per 10 units increments

^d^ Per 1 unit increment

**Supplementary Table 3. Prediction Models for Risk of** **Invasive Mechanical Ventilation (*N*= 80) for Hospitalized Patients with Sepsis**

|  |  |  | **Model 1***  **(Clinical Variables)** | | **Model 2***  **(CT-derived Body Composition Parameters)** | | **Model 3***  **(CT-derived Body Composition Parameters and Clinical Variables)** | |
| --- | --- | --- | --- | --- | --- | --- | --- | --- |
| **Variables** | **Unadjusted Odds Ratio (95% CI)** | ***P* value** | **Adjusted Odds Ratio (95% CI)** | ***P* value** | **Adjusted Odds Ratio (95% CI)** | ***P* value** | **Adjusted Odds Ratio (95% CI)** | ***P* value** |
| Male sex | 1.137 (0.658-1.967) | 0.645 | **-** | **-** | **-** | **-** | **-** | **-** |
| Age | 1.026 (1.003-1.049) | **0.024** | - | - | 1.023 (0.999-1.047) | 0.056 | - | - |
| BMI | 0.992 (0.934-1.054) | 0.795 | **-** | - | - | - | - | - |
| COPD | 1.731 (0.757-3.957) | 0.193 | - | - | - | - | - | - |
| Heart failure | 2.912 (1.711-4.956) | **0.000** | - | - | - | - | - | - |
| Hypertension | 0.753 (0.443-1.280) | 0.294 | - | - | - | - | - | - |
| CKD | 3.299 (1.917-5.696) | **0.000** | 2.210 (1.185-4.122) | **0.013** | - | - | 2.247 (1.211-4.167) | **0.010** |
| Diabetes | 1.081 (0.622-1.880) | 0.782 | - | - | - | - | - | - |
| Chronic liver disease | 4.690 (2.177-10.104) | **0.000** | - | - | - | - | - | - |
| PaO_2_/FiO_2_ | 0.988 (0.984-0.992) | **0.000** | 0.989 (0.986-0.993) | **0.000** |  |  | 0.990 (0.986-0.993) | **0.000** |
| WBC | 1.073 (1.032-1.116) | **0.000** | - | - | - | - | - | - |
| Neutrophil count | 1.091 (1.046-1.139) | **0.000** | 1.065 (1.018-1.115) | **0.007** | - | - | 1.062 (1.015-1.112) | **0.010** |
| Lymphocyte count | 0.519 (0.317-0.849) | **0.009** | - | - | - | - | - | - |
| Hgb | 0.988 (0.977-0.998) | **0.022** | - | - | - | - | - | - |
| PLT | 0.999 (0.996-1.001) | 0.268 | - | - | - | - | - | - |
| D dimer | 1.127 (1.037-1.224) | **0.005** | 1.060 (1.008-1.114) | **0.022** | - | - | - | - |
| TNI | 1.028 (0.945-1.117) | 0.522 | - | - | - | - | - | - |
| ALT | 1.004 (1.000-1.008) | 0.055 | - | - | - | - | - | - |
| AST | 1.006 (1.001-1.011) | **0.023** | 1.005 (0.999-1.010) | 0.078 | - | - | 1.005 (1.000-1.010) | 0.058 |
| GGT | 0.999 (0.995-1.003) | 0.606 | - | - | - | - | - | - |
| ALB | 0.956 (0.914-1.001) | 0.056 | - | - | - | - | - | - |
| Cr | 1.002 (0.999-1.005) | 0.123 | - | - | **-** | **-** | - | - |
| CRP | 1.003 (1.000-1.006) | 0.051 | **-** | **-** | **-** | **-** | **-** | **-** |
| SMA_T4_ ^a^ | 0.944 (0.884-1.008) | 0.087 | **-** | **-** | - | - | - | - |
| SMI_T4_ ^a^ | 0.792 (0.641-0.977) | **0.030** | **-** | **-** | 0.698 (0.548-0.889) | **0.004** | - | - |
| SMA_L1_ ^a^ | 1.015 (0.935-1.102) | 0.725 | **-** | **-** | **-** | **-** | **-** | **-** |
| SMI_L1_ ^a^ | 1.030 (0.800-1.327) | 0.819 | **-** | **-** | **-** | **-** | **-** | **-** |
| SAT_L1_ ^a^ | 1.000 (0.959-1.043) | 0.987 | **-** | **-** | - | - | - | - |
| AC_L1_ ^a^ | 1.241 (0.994-1.548) | 0.056 | **-** | **-** | 1.512 (1.170-1.954) | **0.002** | 1.105 (0.859-1.421) | 0.437 |
| IMAT_L1_^b^ | 0.992 (0.939-1.049) | 0.781 | **-** | **-** | - | - | - | - |

Bold face indicates a *P* value with statistical significance. CT computed tomography; CI confidence interval; BMI body mass index; COPD chronic obstructive pulmonary disease; CKD chronic kidney disease; PaO_2_ arterial partial pressure of oxygen; FiO_2_ fractional oxygen concentration; WBC white blood cell; Hgb hemoglobin; PLT platelet; TNI troponin I; ALT alanine aminotransferase; AST aspartate transaminase; GGT gamma-glutamyl transferase; ALB albumin; Cr creatinine; CRP C-reactive protein; SMA_T4_ skeletal muscle area at T4 level; SMI_T4_ skeletal muscle index at T4 level; SMA_L1_ skeletal muscle area at L1 level; SMI_L1_ skeletal muscle index at L1 level; SAT_L1_ subcutaneous adipose tissue at L1 level; AC_L1_ abdominal circumference at L1 level; IMAT_L1_ intramuscular adipose tissue at L1 level.

^*^ Variables selected through multivariable linear regression with backward elimination (Supplementary Tables 5-7)

^a^ Per 10 units increments

^b^ Per 1 unit increment

**Supplementary Table 4. Prediction Models for Risk of** **Septic shock (*N*= 78) for Hospitalized Patients with Sepsis**

|  |  |  | **Model 1***  **(Clinical Variables)** | | **Model 2***  **(CT-derived Body Composition Parameters)** | | **Model 3***  **(CT-derived Body Composition Parameters and Clinical Variables)** | |
| --- | --- | --- | --- | --- | --- | --- | --- | --- |
| **Variables** | **Unadjusted Odds Ratio (95% CI)** | ***P* value** | **Adjusted Odds Ratio (95% CI)** | ***P* value** | **Adjusted Odds Ratio (95% CI)** | ***P* value** | **Adjusted Odds Ratio (95% CI)** | ***P* value** |
| Male sex | 1.081 (0.624-1.874) | 0.780 | **-** | **-** | **-** | **-** | **-** | **-** |
| Age | 1.032 (1.008-1.056) | **0.008** | - | - | - | - | - | - |
| BMI | 0.949 (0.890-1.012) | 0.113 | **-** | - | 0.848 (0.766-0.939) | **0.002** | - | - |
| COPD | 0.632 (0.231-1.730) | 0.372 | - | - | - | - | - | - |
| Heart failure | 4.537 (2.629-7.830) | **0.000** | 2.910 (1.510-5.605) | **0.001** | - | - | - | - |
| Hypertension | 0.989 (0.585-1.674) | 0.968 | - | - | - | - | - | - |
| CKD | 4.432 (2.553-7.695) | **0.000** | 2.345 (1.178-4.667) | **0.015** | - | - | 3.566 (1.884-6.749) | **0.000** |
| Diabetes | 0.966 (0.549-1.697) | 0.903 | - | - | - | - | - | - |
| Chronic liver disease | 3.110 (1.456-6.641) | **0.003** | - | - | - | - | - | - |
| PaO_2_/FiO_2_ | 0.987 (0.983-0.991) | **0.000** | 0.991 (0.986-0.995) | **0.000** |  |  | 0.987 (0.983-0.992) | **0.000** |
| WBC | 1.064 (1.024-1.105) | **0.002** | - | - | - | - | - | - |
| Neutrophil count | 1.080 (1.036-1.125) | **0.000** | - | - | - | - | - | - |
| Lymphocyte count | 0.523 (0.318-0.858) | **0.010** | - | - | - | - | - | - |
| Hgb | 0.982 (0.971-0.993) | **0.001** | - | - | - | - | - | - |
| PLT | 0.999 (0.996-1.001) | 0.409 | - | - | - | - | - | - |
| D dimer | 1.070 (0.995-1.151) | 0.069 | - | - | - | - | - | - |
| TNI | 0.989 (0.905-1.080) | 0.804 | - | - | - | - | - | - |
| ALT | 1.005 (1.001-1.009) | **0.026** | - | - | - | - | - | - |
| AST | 1.010 (1.004-1.016) | **0.023** | 1.008 (1.001-1.015) | **0.032** | - | - | 1.009 (1.002-1.015) | **0.010** |
| GGT | 1.001 (0.997-1.004) | 0.768 | - | - | - | - | - | - |
| ALB | 0.968 (0.925-1.013) | 0.162 | - | - | - | - | - | - |
| Cr | 1.003 (1.000-1.005) | **0.041** | - | - | **-** | **-** | - | - |
| CRP | 1.002 (0.999-1.005) | 0.126 | **-** | **-** | **-** | **-** | **-** | **-** |
| SMA_T4_ ^a^ | 0.919 (0.858-0.984) | **0.015** | **-** | **-** | - | - | - | - |
| SMI_T4_ ^a^ | 0.742 (0.596-0.923) | **0.007** | **-** | **-** | 0.690 (0.515-0.923) | **0.013** | - | - |
| SMA_L1_ ^a^ | 0.986 (0.906-1.073) | 0.743 | **-** | **-** | 0.962 (0.831-1.115) | 0.609 | **-** | **-** |
| SMI_L1_ ^a^ | 0.964 (0.743-1.249) | 0.779 | **-** | **-** | - | - | **-** | **-** |
| SAT_L1_ ^a^ | 0.997 (0.955-1.041) | 0.891 | **-** | **-** | - | - | 0.933 (0.869-1.002) | 0.057 |
| AC_L1_ ^a^ | 1.282 (1.025-1.603) | **0.029** | **-** | **-** | 2.492 (1.649-3.767) | **0.000** | 1.485 (1.054-2.094) | **0.024** |
| IMAT_L1_^b^ | 1.043 (0.991-1.099) | 0.109 | **-** | **-** | - | - | - | - |

Bold face indicates a *P* value with statistical significance. CT computed tomography; CI confidence interval; BMI body mass index; COPD chronic obstructive pulmonary disease; CKD chronic kidney disease; PaO_2_ arterial partial pressure of oxygen; FiO_2_ fractional oxygen concentration; WBC white blood cell; Hgb hemoglobin; PLT platelet; TNI troponin I; ALT alanine aminotransferase; AST aspartate transaminase; GGT gamma-glutamyl transferase; ALB albumin; Cr creatinine; CRP C-reactive protein; SMA_T4_ skeletal muscle area at T4 level; SMI_T4_ skeletal muscle index at T4 level; SMA_L1_ skeletal muscle area at L1 level; SMI_L1_ skeletal muscle index at L1 level; SAT_L1_ subcutaneous adipose tissue at L1 level; AC_L1_ abdominal circumference at L1 level; IMAT_L1_ intramuscular adipose tissue at L1 level.

^*^ Variables selected through multivariable linear regression with backward elimination (Supplementary Tables 5-7)

^a^ Per 10 units increments

^b^ Per 1 unit increment

**Supplementary Table 5. Multivariable Linear Regression to Build Model 1 (Clinical Variables)-Backward Elimination (Criterion: Probability of F-to-remove ≥ 0.100)**

| **Model 1**  **Clinical Variables** | **Prediction of Admission to Medical Intensive Care Unit** | | | | **Prediction of Invasive Mechanical Ventilation During Hospitalization** | | | | **Prediction of Septic Shock During Hospitalization** | | | | **Prediction of Mortality During Hospitalization** | | | |
| --- | --- | --- | --- | --- | --- | --- | --- | --- | --- | --- | --- | --- | --- | --- | --- | --- |
| **Entered Variables** | **Selected/Removed** | **Standard. *β*** | ***t*** | ***P* Value** | **Selected/Removed** | **Standard. *β*** | ***t*** | ***P* Value** | **Selected/Removed** | **Standard. *β*** | ***t*** | ***P* Value** | **Selected/Removed** | **Standard. *β*** | ***t*** | ***P* Value** |
| Male sex | Removed step 7 | -0.029 | -0.589 | 0.556 | Removed step 6 | 0.031 | 0.600 | 0.549 | Removed step 7 | 0.033 | 0.672 | 0.502 | Removed step 6 | 0.007 | 0.141 | 0.888 |
| Age | Removed step 15 | -0.063 | -1.241 | 0.216 | Removed step 9 | 0.070 | 1.334 | 0.183 | Removed step 9 | 0.072 | 1.432 | 0.153 | Selected predictor | 0.128 | 2.565 | 0.011 |
| BMI | Removed step 13 | -0.048 | -0.951 | 0.343 | Removed step 7 | -0.015 | -0.292 | 0.770 | Removed step 16 | -0.081 | -1.592 | 0.113 | Removed step 12 | -0.065 | -1.323 | 0.187 |
| COPD | Selected predictor | 0.092 | 1.877 | 0.062 | Removed step 14 | 0.068 | 1.336 | 0.182 | Removed step 15 | -0.063 | -1.290 | 0.198 | Selected predictor | -0.082 | -1.731 | 0.085 |
| Heart failure | Selected predictor | 0.088 | 1.728 | 0.085 | Selected predictor | 0.090 | 1.692 | 0.092 | Selected predictor | 0.169 | 3.296 | 0.001 | Selected predictor | 0.200 | 3.895 | 0.000 |
| Hypertension | Removed step 4 | 0.008 | 0.161 | 0.873 | Removed step 17 | -0.083 | -1.642 | 0.102 | Removed step 8 | -0.040 | -0.825 | 0.410 | Removed step 1 | -0.001 | -0.028 | 0.978 |
| CKD | Removed step 1 | 0.039 | 0.773 | 0.440 | Selected predictor | 0.142 | 2.714 | 0.007 | Selected predictor | 0.185 | 3.617 | 0.000 | Selected predictor | 0.223 | 4.532 | 0.000 |
| Diabetes | Removed step 10 | -0.036 | -0.735 | 0.463 | Removed step 4 | -0.007 | -0.145 | 0.885 | Removed step 3 | -0.021 | -0.438 | 0.662 | Removed step 3 | -0.028 | -0.577 | 0.565 |
| Chronic liver disease | Removed step 6 | 0.021 | 0.394 | 0.694 | Removed step 16 | 0.084 | 1.555 | 0.121 | Removed step 6 | -0.009 | -0.163 | 0.871 | Removed step 2 | 0.008 | 0.163 | 0.871 |
| PaO_2_/FiO_2_ | Selected predictor | -0.274 | -5.418 | 0.000 | Selected predictor | -0.299 | -5.676 | 0.000 | Selected predictor | -0.317 | -6.307 | 0.000 | Selected predictor | -0.253 | -4.996 | 0.000 |
| WBC | Selected predictor | 0.160 | 3.107 | 0.002 | Removed step 15 | -0.374 | -1.307 | 0.192 | Removed step 13 | -0.353 | -1.293 | 0.197 | Selected predictor | -0.567 | -2.069 | 0.039 |
| Neutrophil count | Removed step 8 | -0.567 | -1.096 | 0.274 | Selected predictor | 0.136 | 2.572 | 0.011 | Selected predictor | 0.104 | 1.996 | 0.047 | Selected predictor | 0.627 | 2.284 | 0.023 |
| Lymphocyte count | Selected predictor | -0.212 | -4.142 | 0.000 | Removed step 5 | -0.046 | -0.885 | 0.377 | Removed step 5 | -0.042 | -0.841 | 0.401 | Removed step 5 | -0.041 | -0.438 | 0.662 |
| Hgb | Removed step 16 | -0.085 | -1.484 | 0.139 | Removed step 10 | -0.033 | -0.629 | 0.530 | Selected predictor | -0.136 | -2.343 | 0.020 | Removed step 10 | -0.056 | -1.124 | 0.262 |
| PLT | Removed step 5 | -0.011 | -0.206 | 0.837 | Removed step 2 | -0.025 | -0.472 | 0.637 | Removed step 1 | -0.005 | -0.099 | 0.921 | Selected predictor | 0.104 | 2.081 | 0.038 |
| D dimer | Selected predictor | 0.127 | 2.598 | 0.010 | Selected predictor | 0.126 | 2.466 | 0.014 | Removed step 14 | 0.065 | 1.322 | 0.187 | Selected predictor | 0.111 | 2.307 | 0.022 |
| TNI | Removed step 3 | 0.008 | 0.159 | 0.874 | Removed step 12 | 0.048 | 0.896 | 0.371 | Removed step 4 | 0.018 | 0.355 | 0.723 | Removed step 11 | 0.055 | 1.099 | 0.272 |
| ALT | Removed step 2 | 0.037 | 0.744 | 0.457 | Removed step 13 | -0.112 | -1.175 | 0.241 | Removed step 12 | -0.081 | -0.883 | 0.378 | Removed step 7 | -0.042 | 0.467 | 0.641 |
| AST | Removed step 14 | 0.059 | 1.182 | 0.238 | Selected predictor | 0.126 | 2.436 | 0.015 | Selected predictor | 0.158 | 3.240 | 0.001 | Selected predictor | 0.128 | 2.643 | 0.009 |
| GGT | Removed step 9 | -0.021 | -0.416 | 0.678 | Removed step 8 | -0.047 | -0.918 | 0.360 | Removed step 10 | 0.012 | 0.253 | 0.801 | Removed step 8 | 0.027 | 0.551 | 0.582 |
| ALB | Selected predictor | -0.186 | -3.635 | 0.000 | Removed step 11 | 0.003 | 0.058 | 0.954 | Selected predictor | 0.100 | 1.719 | 0.087 | Removed step 9 | 0.003 | 0.057 | 0.955 |
| Cr | Removed step 12 | 0.059 | 1.181 | 0.238 | Removed step 1 | -0.029 | -0.489 | 0.625 | Removed step 2 | -0.020 | -0.353 | 0.724 | Removed step 4 | 0.027 | 0.475 | 0.635 |
| CRP | Removed step 11 | 0.042 | 0.753 | 0.452 | Removed step 3 | -0.025 | -0.447 | 0.655 | Removed step 11 | -0.029 | -0.534 | 0.594 | Removed step 13 | -0.072 | -1.363 | 0.174 |
| Selected predictors in regression model | COPD, Heart failure, PaO_2_/FiO_2_, WBC, Lymphocyte count, ALB, D dimer | | | | PaO_2_/FiO_2_, AST, D dimer, CKD, Neutrophil count | | | | Heart failure, CKD, PaO_2_/FiO_2_, Hgb, AST | | | | PaO_2_/FiO_2_, CKD, Heart failure, AST, Age | | | |
| Durbin-Waston statistic | 1.724 | | | | 1.913 | | | | 1.430 | | | | 1.296 | | | |

BMI body mass index; COPD chronic obstructive pulmonary disease; CKD chronic kidney disease; PaO_2_ arterial partial pressure of oxygen; FiO_2_ fractional oxygen concentration; WBC white blood cell; Hgb hemoglobin; PLT platelet; TNI troponin I; ALT alanine aminotransferase; AST aspartate transaminase; GGT gamma-glutamyl transferase; ALB albumin; Cr creatinine; CRP C-reactive protein.

**Supplementary Table 6. Multivariable Linear Regression to Build Model 2 (CT-derived Body composition)-Backward Elimination (Criterion: Probability of F-to-remove ≥ 0.100)**

| **Model 2**  **Body composition** | **Prediction of Admission to Medical Intensive Care Unit** | | | | **Prediction of Invasive Mechanical Ventilation During Hospitalization** | | | | **Prediction of Septic Shock During Hospitalization** | | | | **Prediction of Mortality During Hospitalization** | | | |
| --- | --- | --- | --- | --- | --- | --- | --- | --- | --- | --- | --- | --- | --- | --- | --- | --- |
| **Entered Variables** | **Selected/Removed** | **Standard. *β*** | ***t*** | ***P* Value** | **Selected/Removed** | **Standard. *β*** | ***t*** | ***P* Value** | **Selected/Removed** | **Standard. *β*** | ***t*** | ***P* Value** | **Selected/Removed** | **Standard. *β*** | ***t*** | ***P Value*** |
| Male sex | Removed step 3 | -0.059 | -0.788 | 0.431 | Removed step 2 | 0.031 | 0.528 | 0.598 | Removed step 1 | 0.023 | 0.279 | 0.781 | Removed step 2 | 0.030 | 0.491 | 0.623 |
| Age | Removed step 6 | -1.327 | -1.022 | 0.186 | Selected predictor | 0.105 | 1.835 | 0.067 | Removed step 3 | 0.061 | 1.043 | 0.298 | Selected predictor | 0.118 | 2.063 | 0.040 |
| BMI | Removed step 7 | -0.120 | -1.439 | 0.151 | Removed step 1 | -0.061 | -0.781 | 0.435 | Selected predictor | -0.167 | -1.993 | 0.047 | Selected predictor | -0.201 | -2.699 | 0.007 |
| SMA_T4_ | Selected predictor | -0.381 | -5.855 | 0.000 | Removed step 3 | 0.047 | 0.274 | 0.784 | Removed step 2 | 0.314 | 0.496 | 0.620 | Selected predictor | -0.235 | -3.559 | 0.000 |
| SMI_T4_ | Removed step 2 | -0.094 | -0.553 | 0.581 | Selected predictor | -0.195 | -3.076 | 0.002 | Selected predictor | -0.227 | -3.071 | 0.002 | Removed step 1 | 0.099 | 0.621 | 0.535 |
| SMA_L1_ | Removed step 5 | 0.122 | 1.310 | 0.191 | Removed step 4 | 0.061 | 0.654 | 0.514 | Selected predictor | -0.470 | -2.180 | 0.030 | Removed step 4 | 0.007 | 0.080 | 0.937 |
| SMI_L1_ | Removed step 1 | 0.081 | 1.018 | 0.309 | Removed step 5 | 0.057 | 0.661 | 0.509 | Selected predictor | 0.410 | 2.061 | 0.040 | Removed step 5 | 0.027 | 0.342 | 0.732 |
| SAT_L1_ | Selected predictor | -0.211 | -2.953 | 0.003 | Removed step 7 | -0.115 | -1.590 | 0.113 | Selected predictor | -0.168 | -1.925 | 0.055 | Removed step 6 | -0.100 | -1.243 | 0.215 |
| AC_L1_ | Selected predictor | 0.397 | 4.844 | 0.000 | Selected predictor | 0.201 | 3.214 | 0.001 | Selected predictor | 0.507 | 5.058 | 0.000 | Selected predictor | 0.394 | 5.188 | 0.000 |
| IMAT_L1_ | Removed step 4 | 0.059 | 0.905 | 0.366 | Removed step 6 | -0.097 | -1.543 | 0.124 | Removed step 4 | 0.086 | 1.326 | 0.186 | Removed step 3 | 0.036 | 0.584 | 0.560 |
| Selected predictors in regression model | SMA_T4_, SAT_L1_, AC_L1_ | | | | Age, SMI_T4_, AC_L1_ | | | | BMI, SMI_T4_, SMA_L1_, AC_L1_ | | | | Age, BMI, AC_L1,_ SMA_T4_ | | | |
| Durbin-Waston statistic | 1.432 | | | | 1.870 | | | | 1.363 | | | | 1.234 | | | |

BMI body mass index; SMA_T4_ skeletal muscle area at T4 level; SMI_T4_ skeletal muscle index at T4 level; SMA_L1_ skeletal muscle area at L1 level; SMI_L1_ skeletal muscle index at L1 level; SAT_L1_ subcutaneous adipose tissue at L1 level; AC_L1_ abdomen circumference at L1 level; IMAT_L1_ intramuscular adipose tissue at L1 level.

**Supplementary Table 7. Multivariable Linear Regression to Build Model 3 (CT-derived Body Composition and Clinical Variables)–Backward Elimination (Criterion: Probability of F-to-remove ≥ 0.100)**

| **Model 3**  **CT-derived Body Composition and Clinical Variables** | **Prediction of Admission to Medical Intensive Care Unit** | | | | **Prediction of Invasive Mechanical Ventilation During Hospitalization** | | | | **Prediction of Septic Shock During Hospitalization** | | | | **Prediction of Mortality During Hospitalization** | | | |
| --- | --- | --- | --- | --- | --- | --- | --- | --- | --- | --- | --- | --- | --- | --- | --- | --- |
| **Entered Variables** | **Selected/Removed** | **Standard. *β*** | ***t*** | ***P* Value** | **Selected/Removed** | **Standard. *β*** | ***t*** | ***P* Value** | **Selected/Removed** | **Standard. *β*** | ***t*** | ***P* Value** | **Selected/Removed** | **Standard. *β*** | ***t*** | ***P* Value** |
| Male sex | Removed step 15 | -0.048 | -0.747 | 0.456 | Removed step 7 | -0.026 | -0.390 | 0.696 | Removed step 7 | 0.011 | 0.157 | 0.875 | Removed step 7 | 0.047 | 0.667 | 0.505 |
| Age | Selected predictor | -0.102 | -2.025 | 0.044 | Removed step 12 | 0.060 | 1.153 | 0.250 | Removed step 5 | -0.015 | -0.287 | 0.774 | Removed step 11 | 0.059 | 1.134 | 0.258 |
| BMI | Removed step 3 | -0.029 | -0.390 | 0.697 | Removed step 8 | 0.033 | 0.428 | 0.669 | Selected predictor | -0.134 | -1.831 | 0.068 | Selected predictor | -0.126 | -1.742 | 0.083 |
| COPD | Selected predictor | 0.083 | 1.718 | 0.087 | Removed step 19 | 0.060 | 1.190 | 0.235 | Selected predictor | -0.107 | -2.218 | 0.027 | Selected predictor | -0.101 | -2.160 | 0.032 |
| Heart failure | Removed step 16 | 0.067 | 1.285 | 0.200 | Removed step 18 | 0.067 | 1.254 | 0.211 | Selected predictor | 0.147 | 2.931 | 0.004 | Selected predictor | 0.205 | 4.166 | 0.000 |
| Hypertension | Removed step 8 | 0.014 | 0.290 | 0.772 | Removed step 22 | -0.083 | -1.625 | 0.105 | Removed step 13 | -0.046 | -0.963 | 0.337 | Removed step 1 | -0.010 | -0.208 | 0.835 |
| CKD | Removed step 2 | 0.025 | 0.497 | 0.62 | Selected predictor | 0.135 | 2.609 | 0.010 | Selected predictor | 0.194 | 3.958 | 0.000 | Selected predictor | 0.202 | 4.226 | 0.000 |
| Diabetes | Removed step 13 | -0.041 | -0.841 | 0.401 | Removed step 1 | -0.013 | -0.256 | 0.798 | Removed step 10 | -0.044 | -0.894 | 0.372 | Removed step 9 | -0.041 | -0.850 | 0.396 |
| Chronic liver disease | Removed step 4 | 0.006 | 0.110 | 0.912 | Removed step 20 | 0.074 | 1.372 | 0.171 | Removed step 11 | -0.025 | -0.491 | 0.624 | Removed step 5 | -0.009 | -0.184 | 0.854 |
| PaO_2_/FiO_2_ | Selected predictor | -0.287 | -5.780 | 0.000 | Selected predictor | -0.299 | -5.746 | 0.000 | Selected predictor | -0.306 | -6.272 | 0.000 | Selected predictor | -0.276 | -5.744 | 0.000 |
| WBC | Selected predictor | 0.164 | 3.265 | 0.001 | Removed step 16 | -0.214 | -0.730 | 0.466 | Removed step 8 | 0.002 | 0.009 | 0.993 | Removed step 6 | 0.024 | 0.476 | 0.634 |
| Neutrophil count | Removed step 18 | -0.730 | -1.413 | 0.159 | Selected predictor | 0.136 | 2.595 | 0.010 | Selected predictor | 0.086 | 1.707 | 0.089 | Removed step 15 | 0.037 | 0.732 | 0.465 |
| Lymphocyte count | Selected predictor | -0.183 | -3.603 | 0.000 | Removed step 5 | -0.021 | -0.399 | 0.690 | Removed step 6 | 0.005 | 0.109 | 0.913 | Removed step 17 | -0.067 | -1.339 | 0.182 |
| Hgb | Removed step 6 | -0.040 | -0.684 | 0.495 | Removed step 14 | -0.018 | -0.329 | 0.742 | Removed step 15 | -0.081 | -1.374 | 0.171 | Removed step 10 | -0.003 | -0.058 | 0.954 |
| PLT | Removed step 14 | -0.047 | -0.925 | 0.356 | Removed step 2 | -0.027 | -0.521 | 0.603 | Removed step 4 | -0.007 | -0.149 | 0.881 | Selected predictor | 0.080 | 1.716 | 0.087 |
| D dimer | Selected predictor | 0.126 | 2.572 | 0.011 | Selected predictor | 0.123 | 2.408 | 0.017 | Selected predictor | 0.085 | 1.762 | 0.079 | Selected predictor | 0.111 | 2.345 | 0.020 |
| TNI | Removed step 5 | -0.020 | -0.403 | 0.687 | Removed step 17 | 0.030 | 0.571 | 0.569 | Removed step 2 | -0.020 | -0.398 | 0.691 | Removed step 13 | 0.047 | 0.947 | 0.344 |
| ALT | Removed step 7 | -0.066 | 0.729 | 0.467 | Removed step 21 | -0.125 | -1.324 | 0.187 | Removed step 14 | -0.103 | -1.150 | 0.251 | Removed step 12 | -0.022 | -0.251 | 0.802 |
| AST | Selected predictor | 0.088 | 1.835 | 0.068 | Selected predictor | 0.143 | 2.818 | 0.005 | Selected predictor | 0.158 | 3.274 | 0.001 | Selected predictor | 0.126 | 2.620 | 0.009 |
| GGT | Removed step 17 | -0.058 | -1.138 | 0.256 | Removed step 6 | -0.051 | -1.004 | 0.316 | Removed step 1 | -0.017 | -0.332 | 0.740 | Removed step 3 | -0.014 | -0.291 | 0.772 |
| ALB | Selected predictor | -0.130 | -2.436 | 0.015 | Removed step 15 | 0.028 | 0.512 | 0.609 | Selected predictor | 0.126 | 2.404 | 0.017 | Removed step 14 | 0.056 | 1.084 | 0.279 |
| Cr | Removed step 1 | 0.037 | 0.743 | 0.458 | Removed step 4 | -0.041 | -0.707 | 0.480 | Removed step 3 | -0.008 | -0.144 | 0.886 | Removed step 4 | -0.016 | -0.297 | 0.766 |
| CRP | Removed step 12 | 0.032 | 0.572 | 0.568 | Removed step 3 | -0.045 | -0.813 | 0.417 | Removed step 12 | -0.049 | -0.910 | 0.363 | Removed step 16 | -0.041 | -0.840 | 0.401 |
| SMA_T4_ | Selected predictor | -0.244 | -3.908 | 0.000 | Removed step 9 | -0.080 | -0.476 | 0.634 | Removed step 9 | 0.251 | 0.464 | 0.643 | Selected predictor | -0.483 | -3.093 | 0.002 |
| SMI_T4_ | Removed step 9 | -0.060 | -0.401 | 0.689 | Selected predictor | -0.140 | -2.463 | 0.014 | Selected predictor | -0.125 | -1.900 | 0.058 | Selected predictor | 0.303 | 2.120 | 0.035 |
| SMA_L1_ | Removed step 11 | 0.066 | 0.795 | 0.427 | Removed step 10 | -0.042 | -0.503 | 0.615 | Selected predictor | -0.424 | -2.284 | 0.023 | Removed step 2 | -0.060 | -0.754 | 0.451 |
| SMI_L1_ | Removed step 10 | 0.035 | 0.502 | 0.616 | Removed step 11 | -0.018 | -0.234 | 0.815 | Selected predictor | 0.316 | 1.829 | 0.069 | Removed step 8 | -0.057 | -0.782 | 0.435 |
| SAT_L1_ | Selected predictor | -0.199 | -2.910 | 0.004 | Selected predictor | -0.126 | -1.950 | 0.052 | Selected predictor | -0.244 | -3.089 | 0.002 | Selected predictor | -0.175 | -2.350 | 0.019 |
| AC_L1_ | Selected predictor | 0.212 | 2.791 | 0.006 | Selected predictor | 0.177 | 2.501 | 0.013 | Selected predictor | 0.361 | 4.124 | 0.000 | Selected predictor | 0.376 | 4.860 | 0.000 |
| IMAT_L1_ | Selected predictor | 0.103 | 1.821 | 0.070 | Removed step 13 | -0.024 | -0.398 | 0.691 | Selected predictor | 0.119 | 2.124 | 0.035 | Removed step 18 | 0.078 | 1.397 | 0.164 |
| Selected predictors in regression model | Age, COPD, PaO_2_/FiO_2_, WBC, AST, ALB, Lymphocyte count, D dimer, SMA_T4_, SAT_L1_, IMAT_L1_, AC_L1_ | | | | PaO_2_/FiO_2_, AST, CKD, Neutrophil count, AC_L1_ | | | | PaO_2_/FiO_2_, AC_L1_, CKD AST, SAT_L1_ | | | | PaO_2_/FiO_2_, CKD, Heart failure, SMA_T4_, AC_L1_ | | | |
| Durbin-Waston statistic | 1.791 | | | | 1.990 | | | | 1.527 | | | | 1.350 | | | |

BMI body mass index; COPD chronic obstructive pulmonary disease; CKD chronic kidney disease; PaO_2_ arterial partial pressure of oxygen; FiO_2_ fractional oxygen concentration; SOFA sequential organ failure assessment; WBC white blood cell; Hgb hemoglobin; PLT platelet; TNI troponin I; ALT alanine aminotransferase; AST aspartate transaminase; GGT gamma-glutamyl transferase; ALB albumin; Cr creatinine; CRP C-reactive protein; SMA_T4_ skeletal muscle area at T4 level; SMI_T4_ skeletal muscle index at T4 level; SMA_L1_ skeletal muscle area at L1 level; SMI_L1_ skeletal muscle index at L1 level; SAT_L1_ subcutaneous adipose tissue at L1 level; AC_L1_ abdomen circumference at L1 level; IMAT_L1_ intramuscular adipose tissue at L1 level.

**A B C**

**
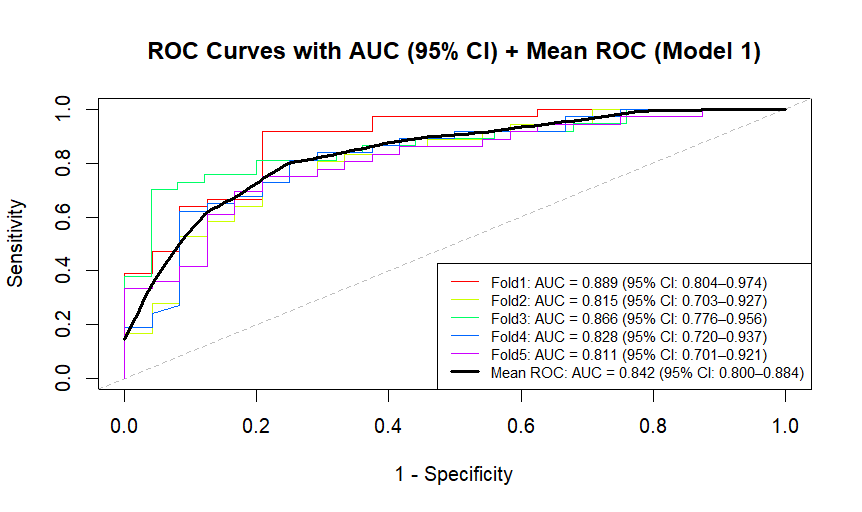

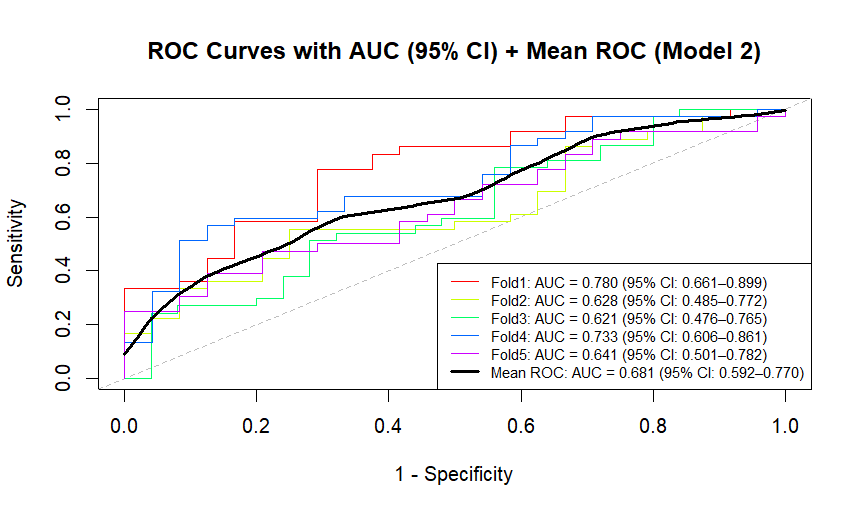

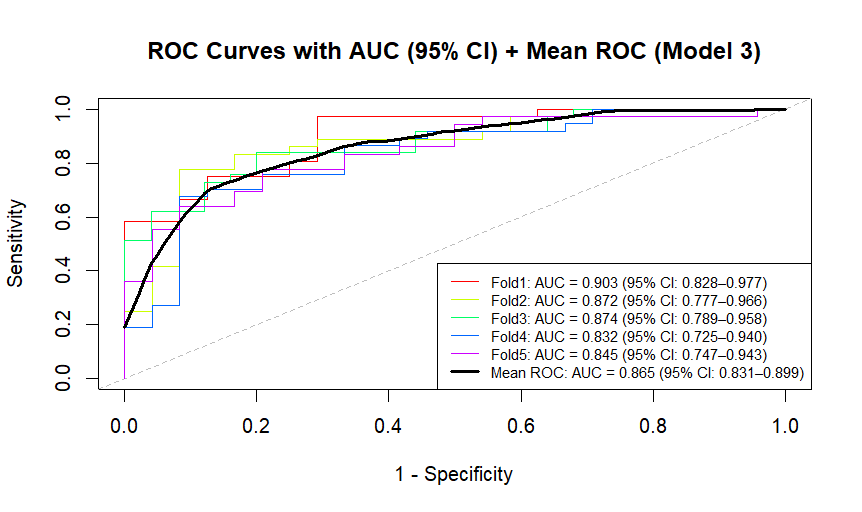
**

**D E F**

**
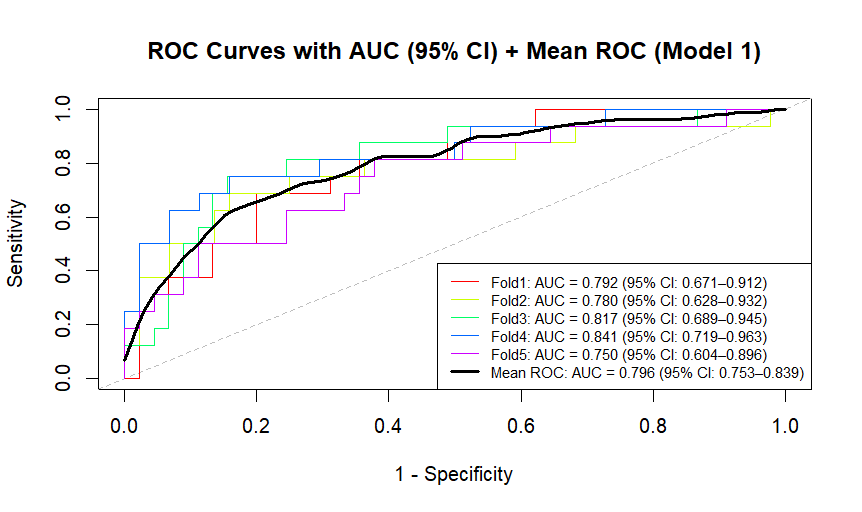

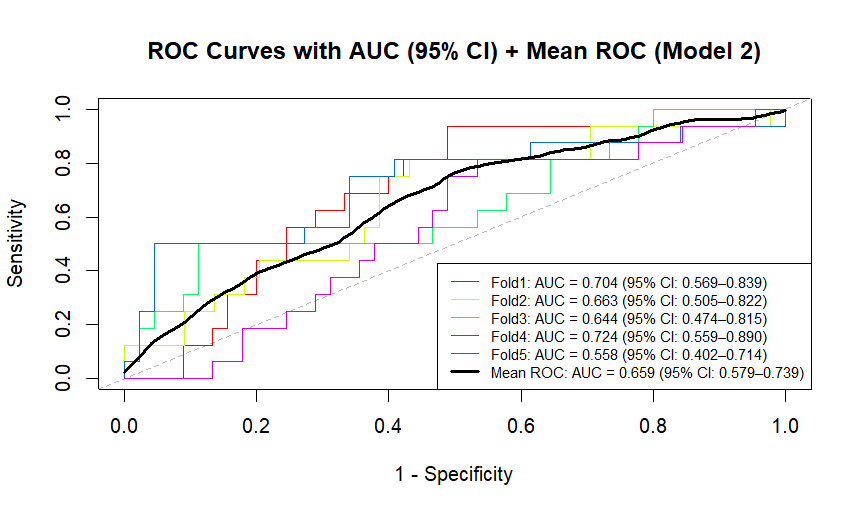

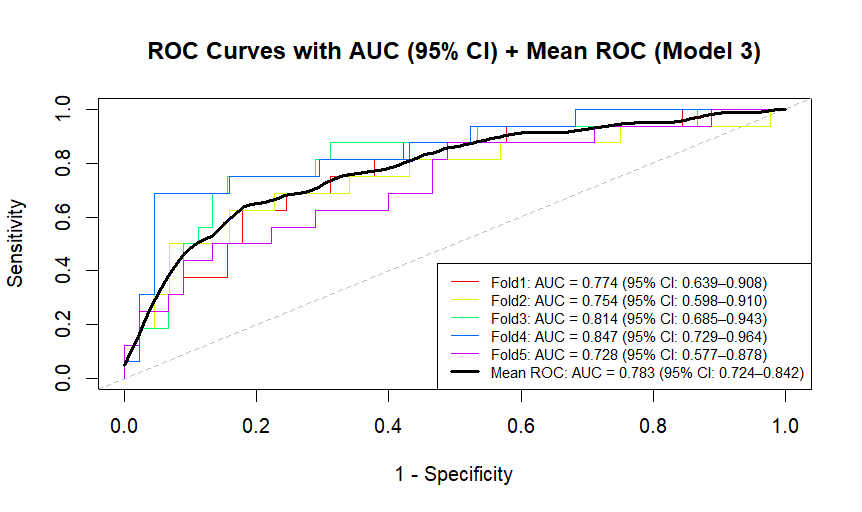
**

**G H I**

**
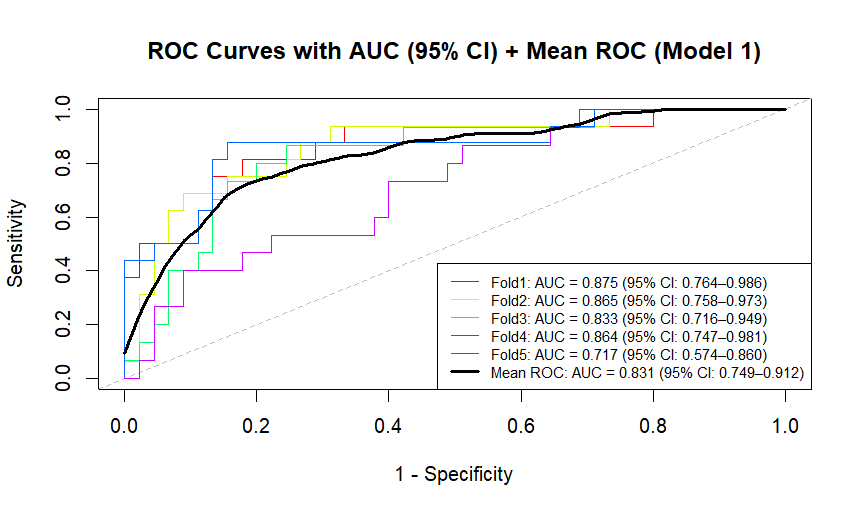

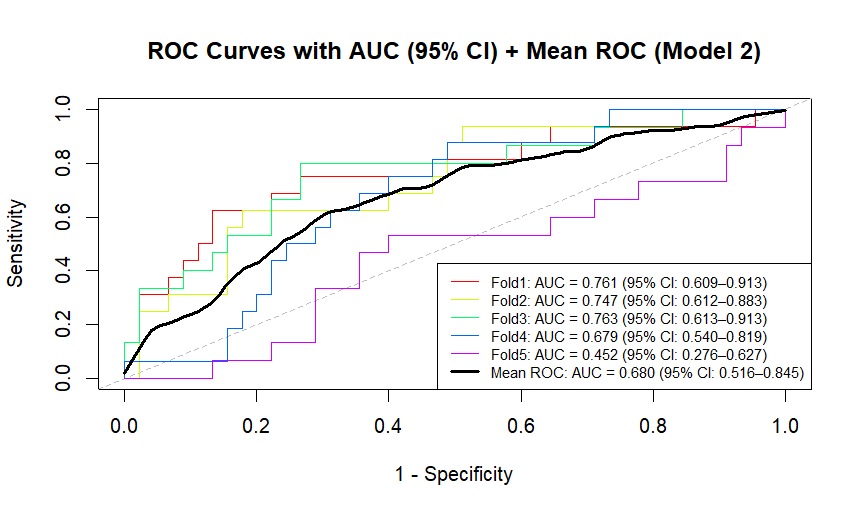

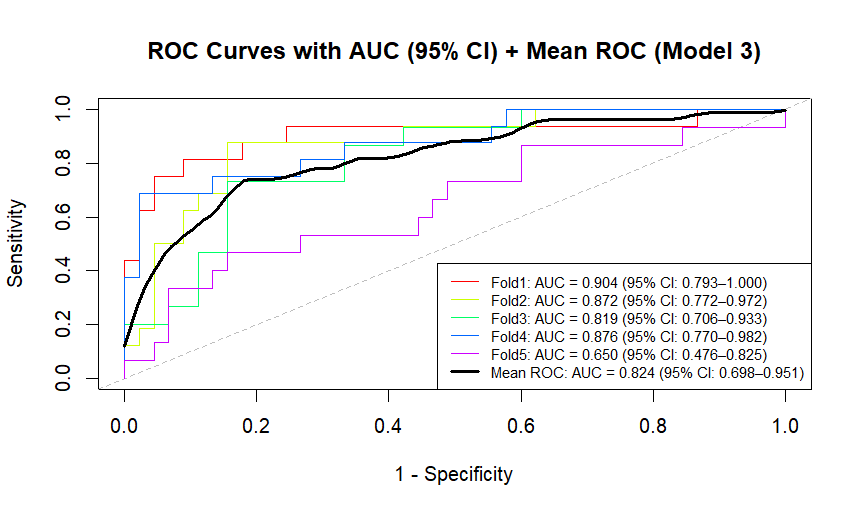
**

**J K L**

**
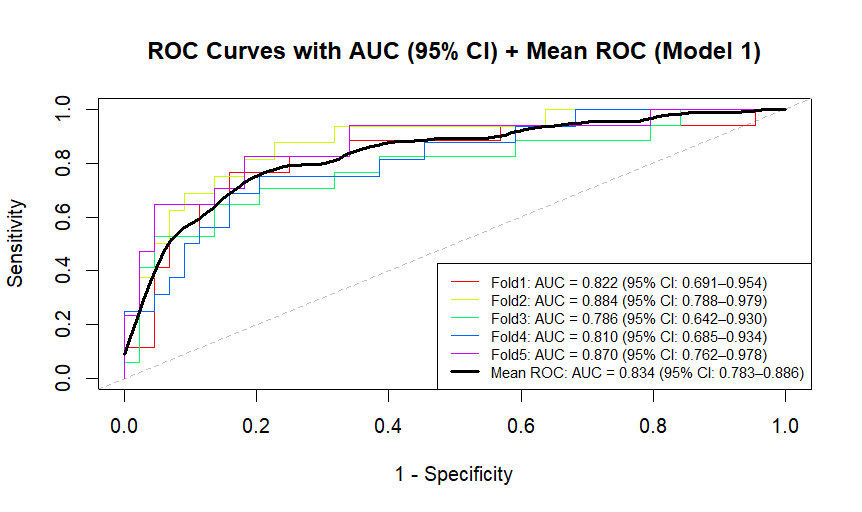

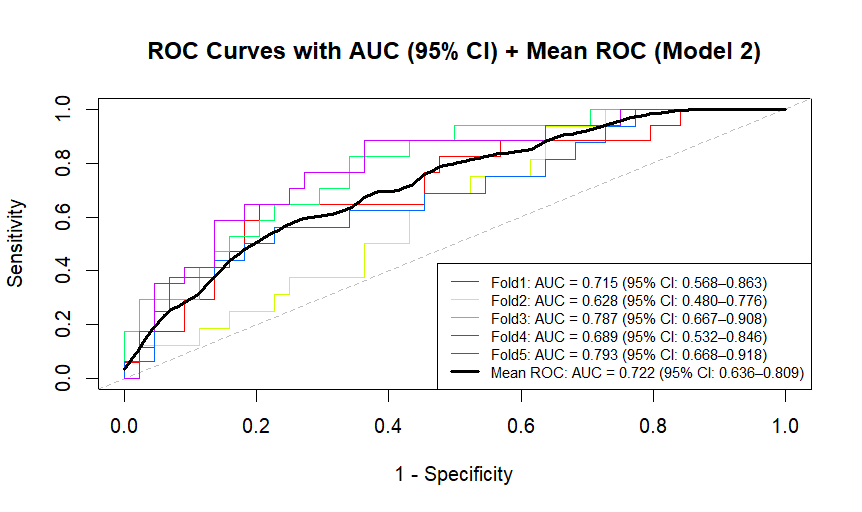

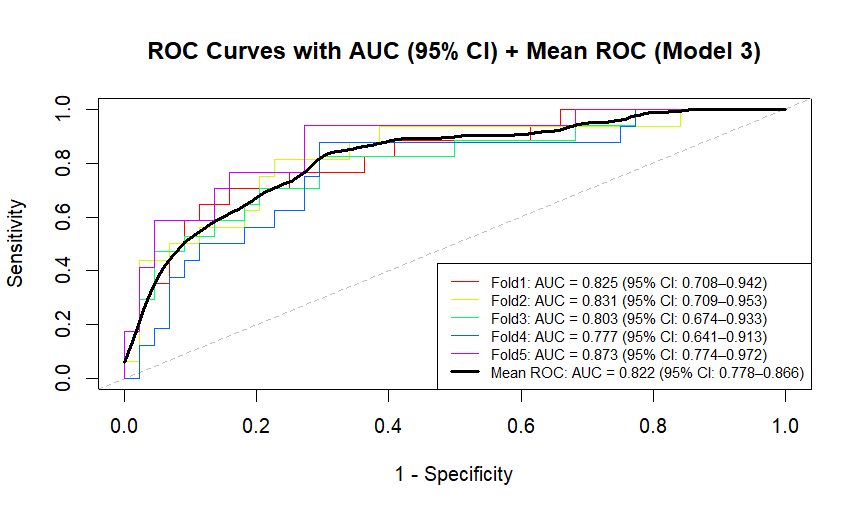
**

**Supplementary Figure 5. The AUC performance of three predictive models subjected to fivefold internal cross-validation (A-C) for medical intensive care unit admission prediction, (D-F) invasive mechanical ventilation prediction, (G-I) septic shock prediction, and (J-L) in-hospital mortality prediction.**

ROC receiver operating characteristic; AUC area under the curve; CI confidence interval.

**Supplementary Table 8. Performance of the three models and SOFA score in predicting adverse events by receiver operating characteristic curve analyses**

| **Prediction** | **Model 1** | |  | **Model 2** | |  | **Model 3** | | **SOFA score** | |
| --- | --- | --- | --- | --- | --- | --- | --- | --- | --- | --- |
|  | **Overall AUC (95%CI)** | **Sensitivity/Specificity** |  | **Overall AUC (95%CI)** | **Sensitivity/Specificity** |  | **Overall AUC (95%CI)** | **Sensitivity/Specificity** | **AUC (95%CI)** | **Sensitivity/Specificity** |
| **MICU admission** | 0.832 (0.786-0.877) | 0.681/0.843 |  | 0.680 (0.618-0.739) | 0.549/0.736 |  | 0.858 (0.819-0.897) ^*^ | 0.703/0.843 | 0.706 (0.659-0.757) | 0.423/0.950 |
| **IMV** | 0.796 (0.738-0.851) | 0.738/0.744 |  | 0.652 (0.583-0.721) | 0.650/0.614 |  | 0.784 (0.718-0.842) | 0.662/0.803 | 0.764 (0.698-0.824) **^#^** | 0.588/0.839 |
| **Septic shock** | 0.826 (0.771-0.877) | 0.744/0.804 |  | 0.680 (0.613-0.750) | 0.590/0.716 |  | 0.817 (0.759-0.871) | 0.782/0.751 | 0.725 (0.658-0.788) | 0.577/0.831 |
| **Hospital Mortality** | 0.822 (0.767-0.875) | 0.795/0.768 |  | 0.720 (0.657-0.778) | 0.687/0.682 |  | 0.815 (0.757-0.868) | 0.735/0.777 | 0.729 (0.664-0.793) | 0.566/0.836 |

SOFA sequential organ failure assessment; AUC area under the curve; CI confidence interval; MICU medical intensive care unit; IMV invasive mechanical ventilation.

^*^ A *P* value with statistical significance Model 3 vs Model 1.

^#^ A *P* value with statistical significance SOFA score vs Model 2.
